# Advancing the service-oriented transition of smart cities: Evaluating the intelligent approval quality

**DOI:** 10.1371/journal.pone.0354902

**Published:** 2026-09-25

**Authors:** Jiaqi Wang, Yali Wang

**Affiliations:** 1 School of Law, Chongqing University, Chongqing, China; 2 School of Law, Taiyuan University of Science and Technology, Taiyuan, Shanxi, China; Zhejiang Shuren University, CHINA

## Abstract

In the context of smart cities development and digital governance, administrative intelligent approval plays an effective and valuable role in government service transformation. However, the explosive application of technology has led to several challenges of approval quality such as inconsistent with due process, weak error correction mechanism of algorithmic decision-making and lagged supervisory regime, which hinder the service-oriented transformation. Therefore, it is essential to evaluate the quality of intelligent approval using rigorous, rational, and universally applicable methods, which may not only enhance the service standards of digital government and advance smart cities development toward maturity, but also contribute to achieving the Sustainable Development Goals. In the stage of evaluation indicator system construction, determining first-level indicators including legality, rationality, satisfaction and supervision by Delphi Method. Then the corresponding 10 secondary indicators and 38 tertiary indicators are progressively expanded based on value guidance, institutional norms, literature research and cases. In the stage of applying, Taiyuan in China is taken as the evaluation object and implemented through Analytic Hierarchy Process and Fuzzy Comprehensive Evaluation. The evaluation results indicated that although the overall quality shows good, there are several potential and prominent problems, including insufficient specific modes, inaccessible the right to a hearing, inadequate staff service, ineffective public supervision and the lack of transparency, and the targeted strategies are proposed for improving the approval quality. Additionally, future research will establish a dedicated evaluation committee, leverage digital platforms more extensively, and strengthen relevant legislation and theoretical support.

## 1. Introduction

### 1.1 Research background

Against the backdrop of the co-evolution of technology and society, the digital public administration has become a defining trend and a core issue in modern nation-building and technological transformation of administrative acts, sparking a global wave of strategic digital government and smart cities initiatives. For example, the “stack” design of the United States, the “government as a platform” concept of the United Kingdom, the “smart country” plan of Singapore, the establishment of the presidential committee for digital platform government in South Korea, and the construction of the online national government service platform in China.

There is no denying that the widespread use of technology has accelerated the process of smart cities planning. However, the explosive expansion of technology has also triggered a potential risk: the transformation of the people-centered service-oriented government into a technology-centered instrumental one. Therefore, it is necessary to pay more attention to the problems in technical governance, improve the quality of digital administration, and guide smart cities to plan in the direction of service-oriented culture.

Administrative approval-an act whereby an administrative authority, upon application by a citizen, legal person, or other organization, and through lawful review, grants permission to engage in a specific activity- has also evolved into intelligent approval in the wave of technological innovation. The intelligent approval refers to a digital governance mode in which certain or all links of the administrative approval process are handled by intelligent technologies such as algorithms [1]. Compared to other digital administrative acts or smart government services, intelligent approval has seen more practical applications due to its broader coverage of public needs, more mature technical conditions, and greater public perceptibility of its service benefits. Furthermore, intelligent approval is not an isolated technological application; it is a core governance module within the digital infrastructure of smart cities. Based on matters models and applicable conditions as its data foundation, supported by intelligent technologies such as expert systems and language/image processing, relying on data sharing and departmental collaboration, and adopting a human–machine collaborative approval process as its mode, intelligent approval forms a smart unit that is seamlessly embedded into the urban digital governance system. Moreover, intelligent approval serves as an important vehicle for the synergistic advancement of the Sustainable Development Goals (SDGs). An empirical study based on 159 countries (2003–2020) demonstrates that intelligent governance, including intelligent approval, has a significant positive impact on all three dimensions of sustainable development [2]. Specifically, in the economic dimension, intelligent approval reduces institutional transaction costs, and allocates resources efficiently. In the social dimension, it breaks through spatial and temporal limitations, enhancing the accessibility of government services. In the environmental dimension, the paperless office practices and reduced physical travel characteristic effectively alleviate resource consumption and environmental pollution.

However, the process of aligning technology with administrative approval inevitably poses distinctive governance challenges. For instance, algorithmic black boxes can affect public trust in administrative organs, algorithmic decision-making restricts the exercise of procedural rights, and the corresponding technical regulatory measures may not have been updated, which not only decreases the quality of intelligent approval, but also interferes with the service-oriented transformation.

Therefore, it is necessary to construct and apply a quality evaluation indicator system to precisely identify the key factors which impact the quality of intelligent approval and analyse the prominent and potential quality problems during practice. Thereby improving approval quality, supporting government service of smart cities planning, and coordinating the ecological and sustainable development of technology and humanities.

### 1.2 Literature review

Regarding the research on the quality of intelligent approval, scholars have contributed many achievements from different perspectives, which also provided meaningful and valuable references for this study.

First, regarding the conceptual positioning of “intelligent approval quality” in the field of public administration. In technical terms, “intelligence” generally refers specifically to “artificial intelligence”, a concept first introduced in 1956 at the Dartmouth Summer Research Project on Artificial Intelligence in the United States [3]. The international standard *ISO/IEC 22989:2022 Information technology-Artificial intelligence-Artificial intelligence concepts and terminology* provides a universally accepted definition of AI: “research and development of mechanisms and applications of AI system (engineered system that generates outputs such as content, forecasts, recommendations or decisions for a given set of human-defined objectives).” Furthermore, this document emphasizes the multi-domain applicability of AI in computer science, data science, humanities, mathematics, and the natural sciences. Within the discipline of public administration, some scholars view intelligence as the use of computer simulations of human intellect to accomplish tasks roughly comparable to human learning, problem-solving, and decision-making [4]. Others argue that intelligence serves as a technical solution that helps public administration become more efficient, accurate, and adaptable, thereby delivering positive benefits to the economy and society [5]. Still others conceive of intelligence as operating through computational intelligence, perceptual intelligence, and cognitive intelligence to simplify public services, optimize public decisions, and expand governmental responsiveness [6]. Overall, although scholars express differences in their definitions of intelligence in the context of public administration, they share a common core: within public administration, the ultimate goal of intelligent applications is the optimization and upgrading of administrative tasks and service capabilities.

Intelligent approval is the specific application of intelligent technologies in administrative approval. Some scholars argue that intelligent approval represents the response of administrative approval reform to the digital era [7], while others consider it a digital administrative model in which particular or all stages of the approval process are handled by technological means in the information age [8]. Against the backdrop of smart city construction, government governance is increasingly reliant on intangible technological management, and the core of a smart city lies in using algorithms, big data, and other intelligent technologies to achieve intelligent government services. Therefore, this article defines the connotation of “intelligent approval” as a governance form in which administrative authorities reshape administrative approval using intelligent technologies, reflecting the service-oriented transformation trend of smart cities construction.

It is worth noting that when scholars explore the disciplinary system of intelligent transformation of public administration, they also often focus on the administrative challenges brought about by intelligence, such as data bias, reduced transparency, regulatory risks, and rigidity of discretionary power [9]. Thus, the next innovation trend in technology-enabled administrative tasks should be how to respond to the high quality development while achieving the generalization of digital government services, thereby defending the rule of law values and service objectives of public administration. As a type of government service within public service, intelligent approval should also pay attention to quality development. The concept of public service quality emerged from the New Public Management movement in the late 1970s and was further developed by the New Public Service theory, which essentially established the goals of high-quality services and quality primacy for public service quality [10]. This is also the value pursuit of intelligent approval quality. Regarding the specific content and implementation paths of public service quality, no consensus has yet been reached in the academic community. For example, some scholars believe that public service quality should focus on three dimensions: institutional legitimacy, perceived effectiveness, and state responsiveness [11]. Others have identified cleanliness as a key factor affecting public service quality and advocate enhancing public perception [12]. Still others, taking the driving role of digital governance as their starting point, argue for strengthening digital infrastructure, deepening targeted digital applications, and promoting coordinated regional development [13]. Consequently, how to improve the quality of intelligent approval has become an urgent issue and a contemporary proposition for the service-oriented transformation of smart cities construction.

From the perspective of practical types, existing research has categorized digital administration based on different criteria, including the discretion-based classification method [14], the substantive impact classification method [15]and so on. Scholars have also developed a classification framework based on German legal theories, which takes artificial intervention as the standard—semi automated administrative acts and fully automated administrative acts [16]. According to synthesized scholarly studies, establishing two levels of intelligent approval based on whether and to what extent the administrative staff participates. In Table 1, the first level is partially intelligent approval, where organs utilize technology for reviewing matters but retain human intervention during the key links; the second level is fully intelligent approval, where the approval system executes tasks according to predefined algorithms and procedures, enabling organs to complete administrative decisions without human intervention.

**Table 1:** Classification of intelligent approval.

Level	Type	Degree of participation		Typical example
		Administrative staff	Technology	
Level-1	Partially intelligent approval	Part participation	Part participation	Teacher qualification certification system
Level-2	Fully intelligent approval	No participation	Full participation	Instant approval

**Fig 1:**
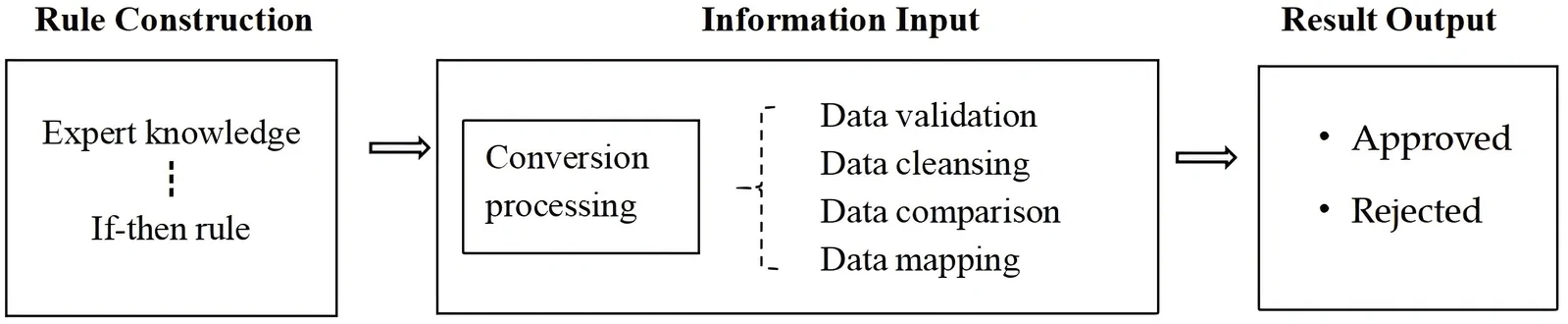
The operating principle of automatic approval.

About the technical principle, scholars have reached a consensus. In digital administration, two primary technical approaches are employed: the rule-based expert system and the machine learning system. The expert system aims to acquire knowledge and experience from human specialists, simulating their logical reasoning and decision-making through “If-then rule” (if A, then execute X; if B, then execute Y), with the underlying logic rooted in deductive reasoning [17]. The machine learning system predicts future trends by analyzing historical data using inductive biases, operating similarly to analogical reasoning [18]. Given the rigorous requirements in administrative approval, which primarily follow deductive reasoning, the core technology of most intelligent approval today has evolved into the rule-based expert system after technical validation and iterative updates. Specifically, the operational mechanism of intelligent approval consists of three phases, including rule construction, information input, and result output, which can be shown in Fig 1. During the rule construction phase, research institutions translate legal regulations into algorithmic “If-then rules” according to administrative requirements, designing intelligent approval systems aligned with procedural standards. The information input phase, the intelligent approval system converts the approval information and materials into recognizable data, and performs a series of operations on the data according to the pre-designed algorithm. Finally, the system makes the decision whether to approve or not [19]. In addition, biometrics, natural language processing, image processing, and other technologies are used as auxiliary to provide technical support for intelligent approval together with the “If-then rule” expert system.

In addition to the above aspects of achievements, existing research also explores the specific coordinated development between intelligent approval and the humanities to improve the quality of intelligent approval. Some scholars have examined the legitimacy of digital administrative acts through frameworks such as Whole-of-Government theory, constitutional norms, legal authorization, and governmental functions [20]. Some focus on algorithmic risks and regulatory measures, including the reliability of algorithmic translation and the justiciability of algorithmic administration [21]. Others emphasize value alignment and rights protection, such as how the technical form of administrative approval should coexist with justice, right of access to information, and oversight mechanisms [22]. And there are also comparative studies trying to further advance institutional frameworks for China’s digital administration, drawing lessons from France’s transparency-centered regulatory model to optimize domestic governance [23]. In addition, some studies have explored its practical effects from the perspective of the rule of law evaluation, the government performance evaluation and database [24].

In summary, relevant research on the phenomenon of technology enabling administrative approval presents multi-dimensional characteristics, has established a robust theoretical foundation, and focuses on how to optimize this digital administrative act and make intelligent approval conform to the construction of government services.

However, most studies remain theoretical, with insufficient exploration of practical implementation. Building upon previous scholarship and adopting a problem-oriented approach, this article focuses on intelligent administrative approval, which is closely tied to public life, and conducts empirical research through the development of an evaluation indicator system, so as to promote the practical transformation of theory, optimize the quality of intelligent approval, and aim to offer a theoretical contribution and empirical reference for the service construction of smart cities.

This article proceeds in five sections. Section 2 sorts the overall practical achievements, typical cases, and prominent quality issues existing in China’s intelligent approval. Section 3 describes evaluation methods, the intelligent approval quality evaluation indicator system design and the corresponding materials. Section 4 describes the applying process of the evaluation indicator system and analyzes the results. Section 5 discusses the significance and highlights in brief, and points out the limitations while proposing specific directions for further work and improvements. Section 6 concludes the core content.

## 2. The status quo and reflection of intelligent approval

With the advent of the big data era, technology has been widely applied in public administration. The development of smart cities is thriving, and intelligent approval practices have been implemented. While technology empowers administration and enables process reengineering, it also transforms the behavioral patterns of administrative organs, giving rise to quality challenges.

### 2.1 The practice landscapes of intelligent approval

According to the *Global AI Index (2024)* released by the UK media outlet Tortoise Media, China ranks second in the world and is among the global leaders in dimensions such as “infrastructure,” “development,” and “government strategy.” This indicates that China’s overall layout in the fields of intelligent governance and digital government transformation has gained international recognition. Moreover, China has a large population and a high volume of approval transactions, making the application of intelligent approval more extensive. More importantly, the processes of intelligent approval in various countries essentially follow the standard workflow of “Application -Acceptance -Decision -Delivery.” Therefore, when this study categorizes and analyzes application scenarios, using China’s intelligent approval practices as examples has good typicality and representativeness.

**Fig 2:**
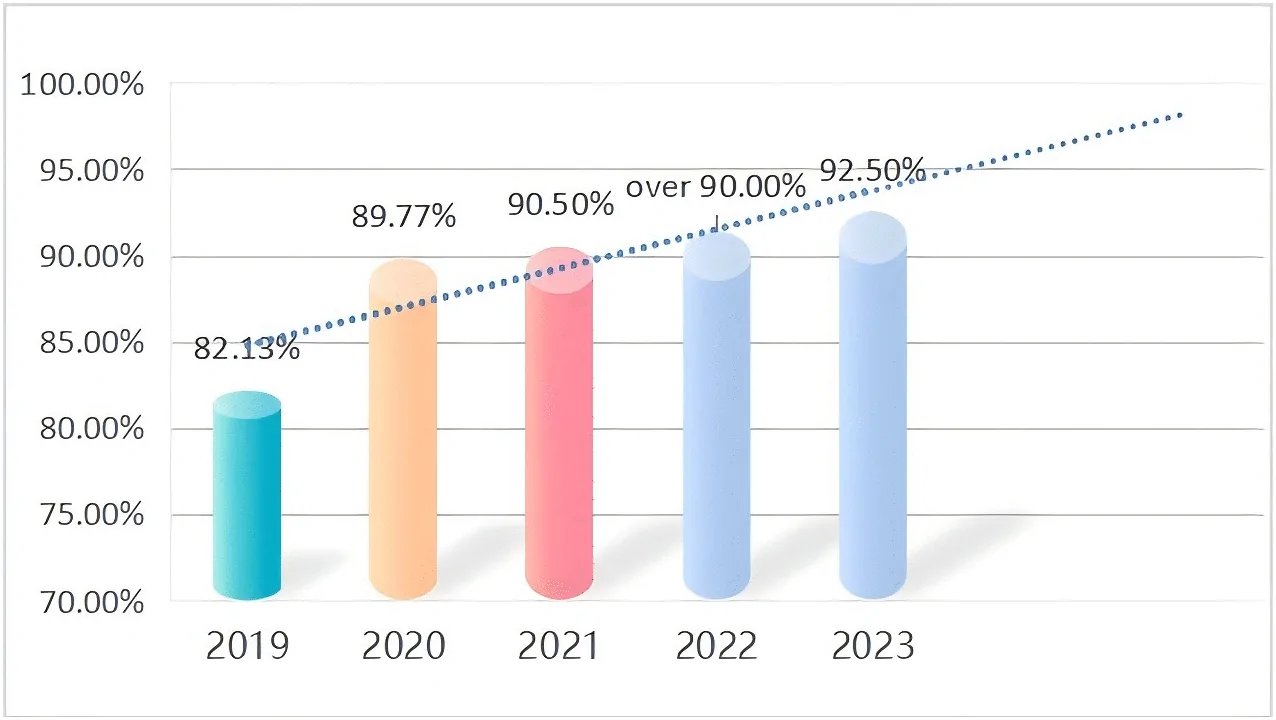
Provincial-level administrative approval matters to achieve online processing and “All in One Go” proportion.

The implementation of intelligent approval in China follows a central-local joint model: At the central level, the National Government Service Platform serves as the backbone, while local governments actively innovate with localized approaches like “Internet + Government Services”, “intelligent approval + Citywide Access”, and “DeepSeek + Digital Governance”. In Fig 2, as reported in the 2023 Digital China Development Report (June 2024), the platform had surpassed 1 billion verified users by 2023, enabling online processing for over 90% of government services nationwide. This progress has driven 92.5% of provincial administrative approvals to achieve online acceptance and the “All in One Go” standard. Looking ahead, the adoption of intelligent approval is expected to maintain its growth trajectory, indicating promising prospects for digital transformation.

Given the diverse features of administrative approval matters, the application of technology in this field shows various degrees. Based on the two-tier classification framework for intelligent approval outlined earlier, presenting typical cases of partially and fully intelligent approval processes to clarify the administrative workflows and operational patterns of different automation types, also to facilitate targeted problem identification and analysis.

#### 2.1.1 The practice landscapes of partially intelligent approval.

In partially intelligent approval scenarios, administrative organs leverage technology to integrate online and offline operations. While slightly less efficient than fully approval systems, this approach allows human involvement to better exercise discretion and provide counterpart with decision-making flexibility. Partially intelligent approvals are primarily applicable to matters requiring substantial discretion, manual verification, or on-site inspections, and those that significantly impact the rights of affected counterparts.

**Fig 3:**
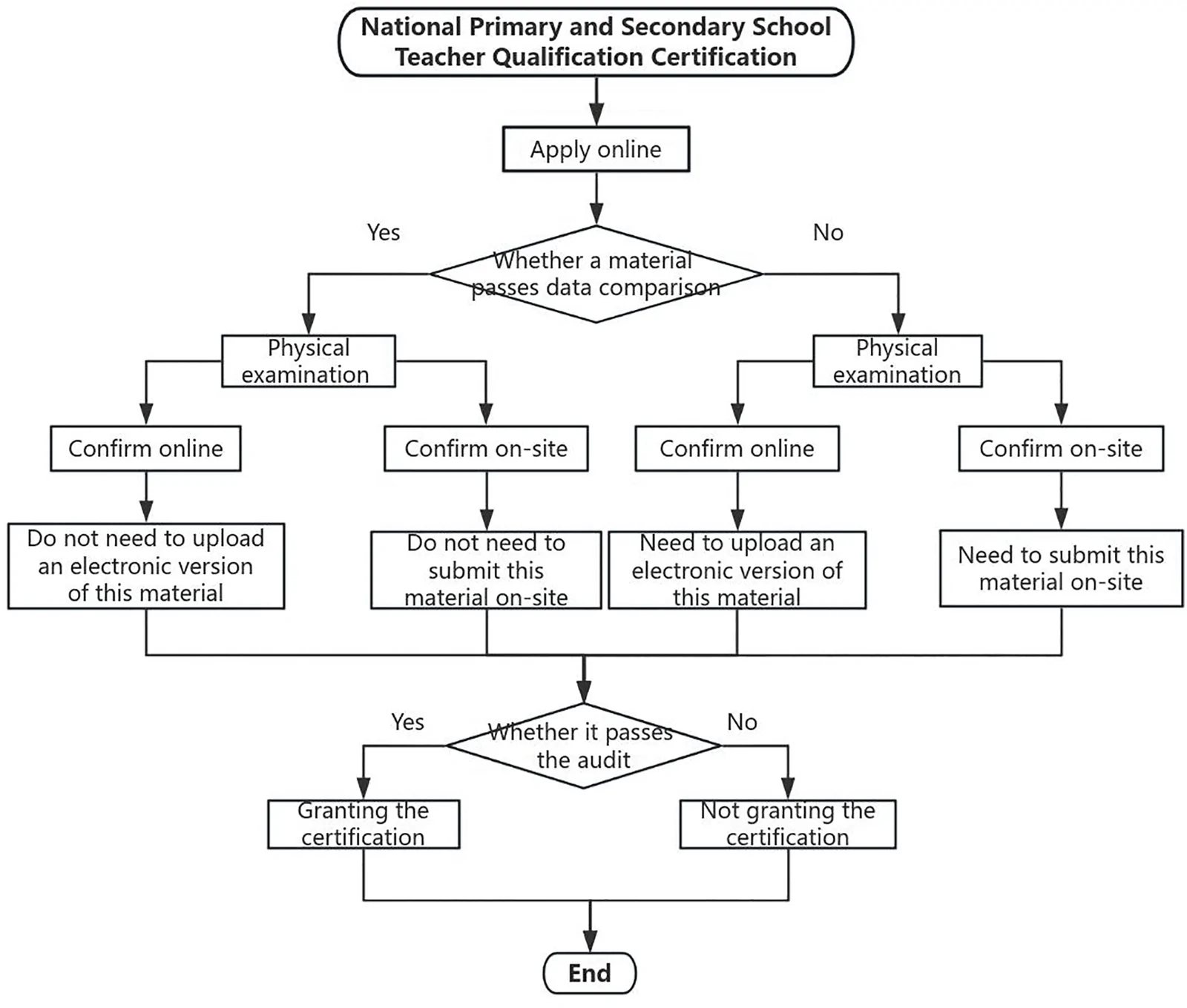
National primary and secondary school teacher qualification process.

For instance, Hebei Province’s Food Business License Approval System handles business operations such as initiation, modification, and renewal through a “human-machine collaboration” model to centrally process food operation applications. This approval system focuses on sorting and uploading information and materials, with the final decision made by administrative staff. Another example is the national teacher certification process for primary and secondary schools, which involves four stages in Fig 3, including online application submission, applicant medical examination, online confirmation or in-person verification, and decision-making, demonstrating effective integration of approval technology with manual processes.

#### 2.1.2 The practice landscapes of fully intelligent approval.

The strength of technology is based on data rather than personal judgment, enabling more accurate and objective decision-making. Consequently, fully intelligent technical processes can ensure more consistent, predictable, and efficient outcomes [25]. Moreover, its workflows eliminate human intervention in administrative procedures, serving as the most effective way to eliminate opportunities for individual rent-seeking behaviors [26], which fosters a healthy administrative environment while reducing oversight costs. Fully intelligent approval systems are primarily applicable to matters with straightforward eligibility criteria, simple procedures, and limited or minimal discretion involved.

**Fig 4:**
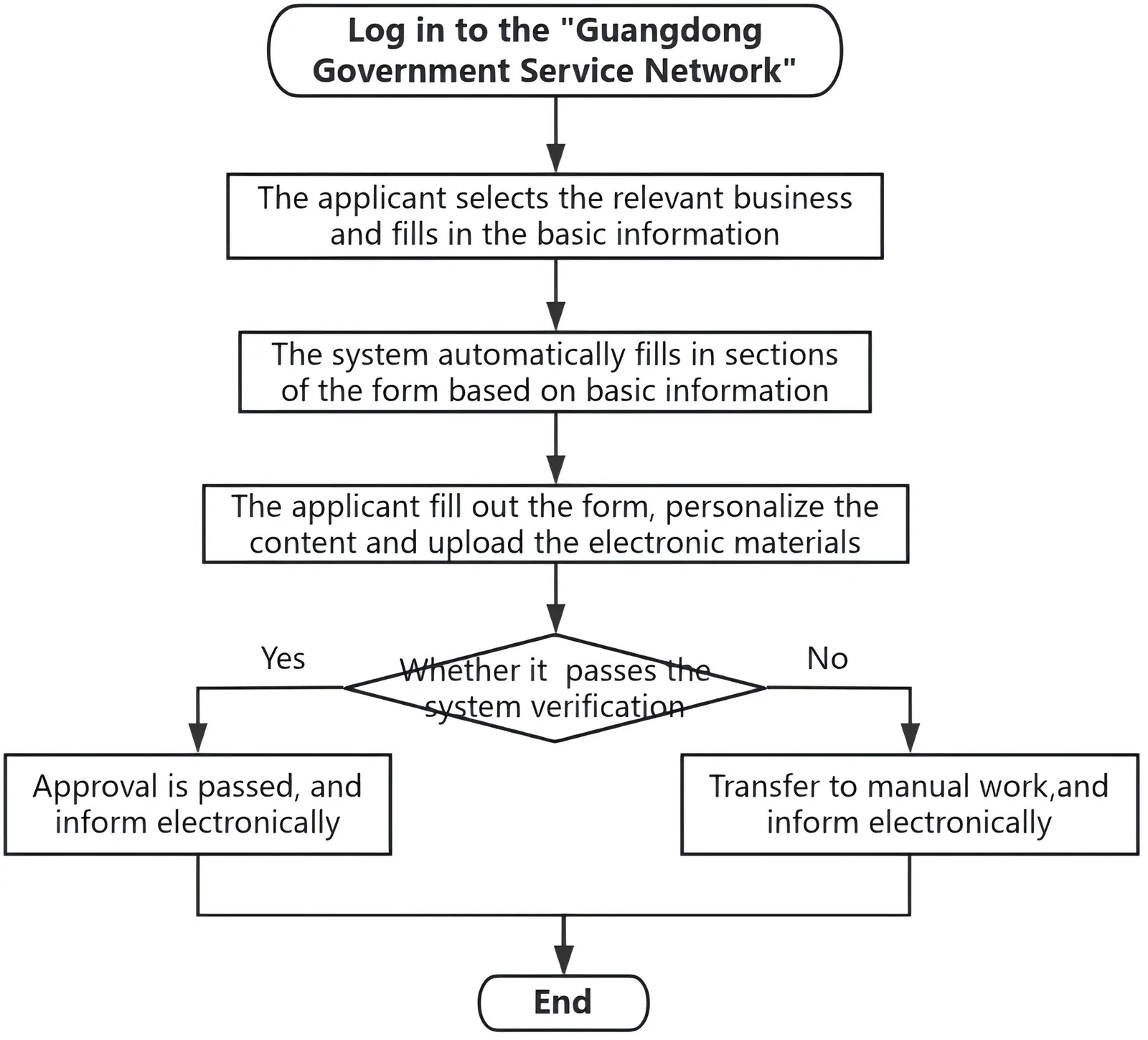
“Instant Approval” system workflow.

A typical local practice is Shenzhen’s “unattended intelligent approval” system implemented in May 2018 for talent introduction and settlement work. Leveraging technology, administrative organs achieved a significant shift from “human approval” to “unattended approval” in administrative approval, which can be shown in Fig 4. This fully intelligent approval method, which requires no administrative staff involvement, delivers near-instant decision-making and is therefore dubbed “Instant Approval”. Similar practices have been implemented in other provinces and cities: In the approval activities of administrative organs in Guangzhou, Nanjing, Chengdu, Henan, and other places, “AI approval” or “intelligent approval” has been implemented by installing self-service all-in-one machines. The machines combine multiple functions such as photography, face recognition, fingerprint collection, and printing. Another notable example is the Market Supervision Administration of Yining City, Ili Prefecture, Xinjiang, which has integrated the DeepSeek large language model into its self-service integrated business registration terminals, which innovatively created a tripartite service system combining “intelligent review, dynamic verification, and closed-loop management”. This approach effectively avoids the applicant carrying and submitting a large number of certification materials, saves the approval cost, and realizes the transformation from “face-to-face approval” to “contactless approval”.

It is not difficult to foresee that with the continuous progress of technology, intelligent approval will continue to explore the digital deposits of rule of law government and provide various forms of digital governance.

### 2.2 Reflection of intelligent approval

While the current administrative legal framework and governance system have become relatively robust, their current structure remains rooted in traditional physical space without adequately considering the unique characteristics of the digital age. Consequently, the anticipated “win-win” scenario between technological integration and administrative approval processes cannot be achieved overnight. The mutual adaptation process between them may lead to law enforcement challenges, which exist in partially intelligent approval systems as well as fully intelligent ones, with some issues being particularly pronounced in fully intelligent environments.

#### 2.2.1 Inconsistent with administrative due process.

Undeniably, technology has improved the efficiency of administrative approval and enhanced the convenience of applications. However, the reshaping of the application process by intelligent approval inevitably brings some impact on the basic procedural bottom line of justice-the principle of due process, and reduces the rationality of administrative approval.

The principle of due process requires administrative actions to meet minimum standards of procedural justice, with its core components being procedural neutrality, openness, fairness, and participation [27]. First, intelligent approval fails to adequately consider the realities faced by “ digitally disadvantaged groups” so that people such as the uneducated or low-educated, disabled groups, and the elderly who cannot keep up with the development of digital services are passively in an unequal position of administrative activity. Moreover, some algorithms lack rigorous design fairness. Data processing habits influenced by administrative staff and developers may reinforce biases as approval processes progress, creating “information cocoons” [28] that overlook elements inconsistent with specific circumstances, thereby leading to non-neutral phenomena like “mechanical judgments” [29]. Particularly in fully intelligent approval, the absence of accessibility for technically disconnected applicants and administrative staff in adjusting technical processing habits exacerbates procedural bias. Such unreasonable differential treatment through algorithmic applications risks evolving into “algorithmic discrimination,” undermining the effective implementation of procedural neutrality [30]. Second, counterparts passively entrust their rights-impacting decisions to algorithms, only able to perceive inputs (application information/materials) and outputs (approval results), but lacking access to algorithmic model structures, data analysis, or running programs [31]. Certain approval systems suspected of having “black box” attributes obscure public disclosures about enforcement processes and standards, eroding trust in administrative organs [32]. Furthermore, the approval conditions contain uncertain legal concepts, which indicates that the approval algorithm needs to translate complex and fuzzy natural language and legal knowledge into simple and accurate codes. The insurmountable “semantic barrier” between legal and technical languages prevents algorithms from fully exercising their discretion, thereby compromising the fairness of fact finding [33]. In the case of “Mr. Wang v. Longli Country Bureau of Land Resources and Planning for Revocation of Mining License,” the court demonstrated concerns about the inadequacy of digital review expertise [34]. Finally, intelligent approval reduces procedures that undermine procedural rights: partially intelligent approval retains administrative participation, preserving the right of statement, right of defence, and right of hearing. However, fully intelligent approval drastically streamlines procedures into a closed loop of “application → instant decision → delivery of decision”, which leaves counterparts no opportunity to exercise rights before finalization. The justice embedded in the old saying “hearing both sides enlightens, hearing one side darkens” will not be achieved [35].

#### 2.2.2 Weak error correction mechanism of algorithmic decision making.

The *Implementation Outline for Building a Government Ruled by Law (2021–2025)*(hereinafter referred to as the *Government Ruled by Law*) emphasizes the need to significantly improve decision-making quality and efficiency, effectively avoiding decision-making errors. That is, administrative organs should ensure efficient law enforcement and accurate outcomes. Efficiency represents the comfort zone of technology, where intelligent approval demonstrates significant advantages in information processing. The streamlined workflow from collection of information and material to review, and then to decision-making and implementation undoubtedly enhances approval efficiency. However, it is precisely due to technological involvement that both partially and fully intelligent approval emergence two distinctive types of decision-making errors: algorithmic flaw and system failures. The foundation of intelligent approval decision-making lies in algorithms that translate normative language into code. The proper operation of these algorithms directly impacts, or even determines, the accuracy of algorithmic decisions. Algorithmic flaw refers to inevitable decision-making errors if the algorithm corresponding to the approval item is defective and incomplete. For instance, code errors in Colorado’s public welfare system in 2007 [36] and Arkansas’s healthcare system in 2016 [37] led to incorrect reductions or rejections of welfare applications. System failures include physical failure, network failure, and operation failure. Physical failure refers to hardware damage caused by failure to repair or replace automation equipment in time, or software problems caused by failure to update the system in time. Network failure results from server communication disruptions caused by sudden surges in website traffic or malicious attacks during peak periods, thereby affecting decision-making accuracy. For instance, in the academic qualification review process for primary and secondary school teacher certification, if the approval website becomes inaccessible, it would prevent real-time communication with the China Higher Education Student Information Website. This would make it impossible to retrieve applicants’ educational records for data verification, leading to erroneous decisions like “academic qualifications not met.” Operation failure is a sudden abnormal operation of the system itself under the premise of reasonable algorithm design. A notable example occurred in September, 2019, when Hangzhou residents encountered widespread consecutive numbers in a housing lottery due to data anomalies where some entries lacked randomization [38]. Similarly, in the case of “Shenzhen Company v. Guangdong Provincial Government,” the approval system made the wrong decision of not accepting the plaintiff due to abnormal circumstances [39] Currently, there remains no systematic institutional framework or practical implementation for correcting such emerging decision-making errors.

#### 2.2.3 The supervisory regime for intelligent approval lags behind.

Administrative supervision aims to find, identify and resolve law enforcement issues while optimizing regulatory practices, thereby ensuring the credibility and execution of administrative acts. The process and outcomes of administrative approval directly impact the rights and interests of counterparts. As a digital governance mechanism, intelligent approval inherently requires corresponding oversight mechanisms to constrain the expanding digital authority. Although China’s administrative supervision framework is relatively systematic, it remains primarily rooted in traditional manual operations without specialized mechanisms tailored for digital governance. For instance, administrative reconsideration-currently the primary internal supervision mechanism-has not liberated the hands of administrative reconsideration personnel by intelligent approval. Instead, the technical transformation or expansion of reconsideration content compels practitioners to possess not only administrative law expertise but also interdisciplinary knowledge. Particularly in the reconsideration involving technical disputes, if there is no participation or advice from technical scholars, or the reconsideration personnel are not trained in automation theory and practice, the reconsideration result may be inappropriate. Additionally, the lack of standardized case-handling guidelines and operational protocols for digital governance further hinders normative reconsideration. Similarly, external oversight mechanisms such as public feedback and administrative complaints require institutional reforms. The UK exemplifies this approach through its Data Protection Officer, a technical advisor who provides expert guidance to administrative organs when handling complaints about algorithmic decision-making systems. China has implemented similar regulations. For instance, *Measures for the Management of Public Data and One Network Office Operation in Shanghai Municipality* stipulates that “public administration and service organs shall establish or designate specialized security management departments with designated security management personnel.” While the role of security management personnel bears some resemblance to the UK’s Data Protection Officer, they differ in two key aspects: first, these personnel are typically administrative staff with relatively weaker technical expertise; second, their responsibilities focus on system security assurance rather than complaint resolution. Additionally, *Opinions on Strengthening the Construction of Administrative Law Enforcement Coordination and Supervision Systems* emphasizes the need to continuously improve supervision mechanisms and innovate supervisory methods. The intelligent approval supervision mechanism requires timely updates and improvements to align with policy guidance.

## 3. Methods for evaluation and materials for the indicator system construction

Evaluation is a systematic quantitative research method that evaluates an object through indicators or standards to reflect its achievement [40]. With the gradual diversification of values and the maturation of methodology, evaluation now encompasses multiple fields such as public policy, corporate risk, educational quality, and legal system. Under the influence of epoch-making momentum, policy guidance, and academic underpinnings, intelligent approval mechanisms have already established the necessary technical prerequisites ex-ante and gained extensive practical traction in-process. In the next stage of development, priority will be given to advancing post-implementation evaluation efforts, utilizing a quality evaluation mechanism that is quantifiable, traceable, feedback-oriented, and capable of targeted improvements to optimize intelligent government services. Therefore, constructing a quality evaluation indicator system suitable for intelligent approval is a critical task in the transition of intelligent approval from an efficiency-first paradigm to a quality-first paradigm. Moreover, through quality evaluation, intelligent approval can engage in “self-reflection” and “self-renewal,” while simultaneously providing more robust and sustained protection for the civil rights and the values of administrative rule of law. This will better align with the requirements of smart city maturity models across multiple dimensions—such as availability, accessibility, safety and security, and quality of service—thereby driving smart cities from the construction phase toward a mature operational phase [41].

### 3.1 Evaluation methods

The methods of this study are Delphi Method (Delphi), Analytic Hierarchy Process (AHP), and Fuzzy Comprehensive Evaluation (FCE). Among them, the Delphi Method is used at the stage of constructing the intelligent approval quality evaluation indicator system, AHP and FCE are mainly used at the evaluation indicator system applying stage.

The combined methodological framework of “Delphi-AHP-FCE” employed in this study possesses a solid foundation of global generalizability. This generalizability does not stem from adherence to specific contexts, but rather from the universality and transferability of its core logic. The framework addresses a three-dimensional dilemma in enhancing intelligent approval quality: First, how to quantify or standardize the vague concept of “quality”? Intelligent approval involves multiple value dimensions—including efficiency, fairness, and service experience—where different stakeholders may hold divergent perceptions of “quality”. Scientific methods are needed to transform vague subjective perceptions into quantifiable objective standards. Second, how to determine the relative importance of sub-factors within the quantified quality metrics? While multiple factors influence quality, their significance varies. Reasonable methods are required to clarify the relative importance of each factor, so as to highlight the main factors influencing quality. Third, how to accurately identify quality problems? Intelligent approval involves a wide range of matters, each with different applicable conditions and procedures, and the quality problems encountered also vary. It is necessary to precisely pinpoint the key bottlenecks of each smart approval matter. These three challenges are not unique to any particular country or region but represent common obstacles in global intelligent approval quality improvement. Consequently, the empirical framework of “Delphi-AHP-FCE” constructed to solve these common problems has the potential for generalizability across different institutional environments and cultural backgrounds. These three methods are specifically designed to address the above three dilemmas: the Delphi leverages expert wisdom to quantify “quality” and screen key indicators; the AHP clarifies the relative weights of each indicator; and the FCE focuses on various quality issues in the form of scores. The organic integration of the three methods forms a complete chain of “indicator establishment-weight clarification-problem identification”, providing a universal and cross-situational empirical research approach for improving intelligent approval quality. This holds replicable reference value for the service-oriented transformation of smart cities globally.

Additionally, the three types of empirical research described above all involve anonymous evaluations of objective subjects by human participants, without any psychological or physical experiments on the participants. All participants are adults with full civil capacity, possessing the necessary qualifications and ability to participate in these empirical research. Prior to commencing these empirical research, researchers provided all participants with a corresponding Informed Consent Form. Each Informed Consent Form clearly stated essential information, including the research purpose, research procedures, duration of participation, potential risks, and anonymity protection. Each Informed Consent Form specifically emphasized the right to voluntary participation and the right to withdraw at any time. The method for obtaining participant consent is anonymous written consent. Specifically, researchers only proceeded with the empirical research (Delphi/AHP/FCE) after the participants personally checked a box confirming: “I have fully understood the Informed Consent Form and voluntarily agree to participate.” The researchers did not engage in any form of inducement, coercion, or information concealment regarding the participants’ act of checking the box.

#### 3.1.1 Determine the first-level indicators by the delphi method.

The Delphi Method, a professional inquiry technique renowned for its anonymity and scientific rigor, has been widely adopted by scholars for indicator development. In general, this method requires 2–3 rounds of survey to determine the indicators, which mainly involve data and calculations of the Coefficient of Expert Authority (Cr), the Kendall’s Coefficient of Concordance (Kendall’s W), and the Coefficient of Variation (CV). In this study, since intelligent approval involves multiple disciplines such as law, management, sociology, and technology, the scholars to be invited should meet the requirements of multiple disciplines. Moreover, since intelligent approval is highly practical, experts with practical experience should also be invited.

The Cr value reflects an expert’s self-assessment, determined by two key factors: the Coefficient of Judgment Basis (Ca) and the Coefficient of Familiarity (Cs). The Ca of this study is assessed based on four factors: practical experience, theoretical knowledge, awareness of peer work, and intuition. The Cs of this study is rated on a five-point scale: unfamiliar, slightly familiar, moderately familiar, familiar, and very familiar. The value of Cr is between 0 and 1. The larger the value, the better the authority of experts. Experts are generally considered authoritative when the Cr exceeds 0.7. The calculation of Cr is as follows:


$$\begin{array}{c} Cr=\frac{Ca+Cs}{\text{2}} \end{array}$$ (1)

The Kendall’s W is a statistic used to assess the degree of overall consistency among multiple judges evaluating a set of objects. The value of Kendall’s W is between 0 and 1. The larger the value, the better the overall consistency. The overall consistency is considered valid when the Kendall’s W exceeds 0.3. The calculation is as follows (where *S* is the sum of squared deviations between each object’s total rating and the mean of all total ratings, *K* is the number of experts, and *N* is the number of objects.):


$$\begin{array}{c} Kendall\prime s\;W=\frac{S}{\;\frac{\text{1}}{\text{12}}\;K^{\text{2}}\;(N^{\text{3}}-N)} \end{array}$$ (2)

The CV is used to measure the degree of dispersion of expert opinions on a particular issue. The value of CV is between 0 and +∞. The smaller the value, the better the particular consistency. The particular consistency is considered good when CV is below 0.25. The calculation is as follows (where is the standard deviation of the *j-*th indicator, and $M_{j}$ is the arithmetic mean of the *j-*th indicator):


$$\begin{array}{c} {CV}_{j}=\frac{\partial_{j}}{M_{j}} \end{array}$$ (3)

#### 3.1.2 Determine the indicators weights by AHP.

AHP is a weighting calculation method that systematically categorizes and prioritizes elements through hierarchical modeling. By establishing a hierarchical structure model (i.e., an indicator system), constructing judgment matrices, calculating weight vectors, and conducting consistency checks, it clarifies the relative proportions of indicators and identifies key factors. Particularly effective for handling complex decision-making problems involving multi-level indicators, this method has been widely adopted by scholars for metric weighting calculations. Therefore, this study employs AHP to determine the weights of the evaluation indicator system.

Firstly, inviting experts including scholars from various disciplines and personnel with practical experience to compare the elements of each level of indicators according to the scale method from 1 to 9 in Table 2.

**Table 2:** Comparison scale table of elements in the judgment matrix.

Meaning of scale	Specific value
The former element i is as important as the latter element j.	a_ij_ = 1
Compared with the latter element j, the former element i is slightly more important than j.	a_ij_ = 3
Compared with the latter element j, the former element i is obviously more important than j.	a_ij_ = 5
Compared with the latter element j, the former element i is strongly more important than j.	a_ij_ = 7
Compared with the latter element j, the former element i is absolutely more important than j.	a_ij_ = 9
It means that the importance of element i and element j is between the above judgments.	a_ij_ = 2,4,6,8
If the relative importance scale of element i and element j is aij, then the relative importance scale of element j and i is aji = 1/ aij	count backwards

By judging each pair of elements in the matrix according to the scale in the above table, we can get the n-order comparison judgment matrix *A:*


$$\begin{array}{c} A_{n\times n}=[\begin{array}{cccc} a_{\text{11}} & a_{\text{12}} & a_{\text{1}..} & a_{\text{1}n}\\ a_{\text{21}} & a_{\text{22}} & a_{\text{2}..} & a_{\text{2}n}\\ a_{..} & a_{..} & a_{..} & a_{..}\\ a_{n\text{1}} & a_{n\text{2}} & a_{n..} & a_{nn} \end{array}] \end{array}$$ (4)

Secondly, the relative weight of each level indicators are calculated by sum method. The calculation steps are as follows:

Step1, get the judgment matrix *A*.
Step 2, each column vector of the matrix *A=(a*_*ij*_*)n × n* is normalized to obtain *B=(b*_*ij*_*)n × n*.

$$\begin{array}{c} b_{ij}=\frac{a_{ij}}{\sum_{i=\text{1}}^{n}a_{ij}} \end{array}$$ (5)

Step 3, add the row vectors of the normalized matrix B.

$$\begin{array}{c} M_{i}=\sum_{j=\text{1}}^{n}b_{ij} \end{array}$$ (6)

Step 4, the vector M is normalized.$\left[\begin{array}{c} \text{M1}\\ \text{M2}\\ \ldots \\ {\text{M}}_{\text{n}} \end{array}\right]$

$$\begin{array}{c} W_{i}=\frac{M_{i}}{\sum_{i=\text{1}}^{n}M_{i}} \end{array}$$ (7)

Step 5, the eigenvector is obtained ${\text{W}}_{\text{i}}=[\begin{array}{c} \text{W1}\\ \text{W2}\\ \ldots \\ {\text{W}}_{\text{n}} \end{array}]$, that is, the relative weight.

Thirdly, testing the consistency. When CR < 0.1, the judgment matrix is considered to have satisfactory consistency and the relative weight is acceptable. The calculation steps are as follows:

Step 1, calculate the maximum eigenvalue λmax.

$$\begin{array}{c} \lambda_{max}=\frac{\text{1}}{n}\sum_{i=\text{1}}^{n}\frac{{[AW]}_{i}}{W_{i}} \end{array}$$ (8)

Step 2, calculate the Consistency Index(CI).

$$\begin{array}{c} CI=\frac{\lambda_{max}-n}{(n-\text{1})} \end{array}$$ (9)

Step 3, calculate the Consistency Ratio(CR).

$$\begin{array}{c} CR=\frac{CI}{RI}=\frac{\lambda_{max}-n}{(n-\text{1})RI} \end{array}$$ (10)

Among them, RI is the Randomness Index, and its specific value is shown in the following Table 3.

**Table 3:** Average random consistency index RI value of the judgment matrix.

Matrix order	1	2	3	4	5	6	7	8	9	10	11	12
**RI**	0	0	0.52	0.89	1.12	1.26	1.36	1.41	1.46	1.49	1.52	1.54

#### 3.1.3 Implementing evaluation and calculating results by FCE.

FCE, commonly used in assessment work, is frequently combined with AHP to evaluate practical implementation of indicators at various levels and make comprehensive decisions. Widely adopted by scholars for evaluation research due to its clear results, credible conclusions, and systematic advantages, this method demonstrates strong applicability in practical applications. More importantly, the FCE has distinct advantages in comprehensive evaluations that involve subjectivity and fuzziness. The evaluation object of this study is the quality of intelligent approval, which inevitably involves concepts with difficult-to-define boundaries at the value level, such as “transparency” and “fairness,” making them hard to measure directly with precise numerical values. In the context of FCE, evaluators use a graded evaluation approach, which is more aligned with the cognitive habits of fuzzy concepts and also helps reduce the participation burden on evaluators. In contrast, other evaluation methods, such as the weighted arithmetic mean and the TOPSIS method, require that indicators be quantified with precise numerical values and conform to certain distributions, which does not match the characteristics of the evaluation object of this study.

First, on the premise of constructing the index evaluation system and obtaining the indicators weights, the evaluation grade set is established according to different criteria and score ranges. In this study, as shown in Table 4, the set is built as {Excellent, Good, Average, Poor, Very Poor}, and each evaluation grade corresponds to a score.

**Table 4:** Evaluation grade score range.

Evaluation Criterion	Excellent	Good	Average	Poor	Very Poor
**Mid-value**	90	70	50	30	10
**Score ranges**	[100, 80]	(80, 60]	(60, 40]	(40,20]	(20, 0]

Second, inviting evaluators to evaluate the indicators according to the evaluation grade set. In this study, since the evaluation of intelligent approval involves not only the applicant’s own experience, but also the expertise, the evaluators should include both the public and the experts.

Third, establishing the fuzzy relationship matrix. After quantifying the indicators, the membership degree of the *i*-th factor to the *j*-th evaluation grade is obtained. Here, the membership degree, F, is defined as the proportion of evaluators who assign the *j*-th grade to the *i*-th factor out of the total number of evaluators. Thus the fuzzy relationship matrix is established:


$$\begin{array}{c} F_{ij}=[\begin{array}{cccc} f_{\text{11}} & f_{\text{12}} & \ldots & f_{\text{1}n}\\ f_{\text{21}} & f_{\text{22}} & \ldots & f_{\text{2}n}\\ \ldots & \ldots & \ldots & \ldots \\ f_{m\text{1}} & f_{m\text{2}} & \ldots & f_{mn} \end{array}] \end{array}$$ (11)

Forth, calculating the membership degree of each indicators layer. This study uses weighted average operator model to multiply the weight *W*_*i*_ with fuzzy matrix *F*_*ij*_ to obtain the membership degree matrix *Z*_*ij*_ of each indicators layer.


$$\begin{array}{c} Z_{ij}=W_{i}*F_{ij}={[z}_{ij}^{\text{1}}\;z_{ij}^{\text{2}}\;z_{ij}^{\text{3}}\ldots {\;z}_{ij}^{n}] \end{array}$$ (12)

Fifth, calculating the evaluation results. The final fuzzy evaluation results *Y*_*ij*_ of each level indicators is obtained by multiplying the membership degree of each indicator with the corresponding evaluation grade set.


$$\begin{array}{c} {\;Y}_{ij}=\left[z_{ij}^{\text{1}}\;z_{ij}^{\text{2}}\;z_{ij}^{\text{3}}\ldots {\;z}_{ij}^{n}\right]\times {\left[\text{90 70 50 30 10}\right]}^{T} \end{array}$$ (13)

### 3.2 Overview of materials for the quality evaluation indicator system construction

It should be noted that since the construction of the intelligent approval quality evaluation indicator system involves the design of various indicators, this stage has a clear source of materials. However, the application of the indicator system is the evaluation of the object by the evaluator, so there is no discussion on the source of materials in the applying stage.

#### 3.2.1 The new public service theory provides value guidance.

The new public service theory represents a critical evolution of the new public management theory, prioritizing human-centered values. Under the guidance of new public management theory, administrative approval emphasizes the government’s central role in social governance. However, the government’s “steering” function and the “customer-oriented” principle have led to a government-centered approach in procedural design. In contrast, the new public service theory stresses that the government’s role is “service” rather than “steering,” advocating for a people-centered approach that prioritizes the interests of citizens [42].

Specifically, the new public service theory advocates intelligent approval to optimize administrative procedures through technical support. This technology-driven approach embodies dual objectives: driving digital transformation and clarifying service positioning. Administrative approval has evolved through three phases: from purely manual processes to “Physical Halls + Dedicated Network,” then to the “Internet + Government Services” model, introducing a new online remote way of fully or partially non-contact procedures [43]. Technology should always play a service role in human-machine interaction with broad accessibility. For instance, “Mobile Approval” enables real-time monitoring of progress, allowing applicants to track updates online. In terms of process optimization, the new public service theory advocates prioritizing user needs. The “One-stop” online approval facilitates seamless interdepartmental communication, providing centralized services. For example, the Administrative Approval Service Bureau of Huimin County, Binzhou City, Shandong Province focuses on the “Full Life Cycle” through a unified interface where applicants can complete document preparation, submission, and view acceptance/review/notifications. Similarly, the National Government Service Platform categorizes services by province into sections like “Civil Livelihood Support,” “Enterprise Assistance,” and “Employment & Entrepreneurship Services,” containing diverse approvals including social security applications, tech enterprise certifications, and electronic business license processing, enabling users to quickly locate required approval matters. intelligent approval implements the practical requirements of the new public service theory. The improvement of its quality should continue to promote technology upgrading and process optimization, and focus on improving the service experience of counterparts.

#### 3.2.2 Legal documents offer fundamental compliance.

On the one hand, evaluation-related documents provide overall reference frameworks. As a crucial component of digital governance and modernization of the national governance system, law enforcement evaluation plays a pivotal role. *Guiding Opinions of the State Council on Strengthening the Building of a Digital Government* (hereinafter referred to as the *Digital Government*) has repeatedly emphasized the establishment of an evaluation indicator system for digital government, while the *Government Ruled by Law* explicitly requires administrative organs to conduct evaluation to ensure visible outcomes and sustainable good governance. These documents collectively offer strategic support at the macro level. Furthermore, evaluation research reports on implementation of the outline documents and post-legislative evaluation conducted by institutions such as the Financial and Economic Committee of the National People’s Congress (NPC), the Legislative Affairs Commission of the NPC Standing Committee, and State Council departments have established framework guidelines for selecting targets, methodologies, and procedures in intelligent approval evaluation.

On the other hand, documents related to intelligent approval provide concrete references. At the level of laws and regulations, although the *Administrative License Law* does not directly address intelligent approval, Article 33 conveys a supportive and encouraging stance: administrative organs should promote e-government. During construction, strict adherence to the law’s principles of transparency, fairness, non-discrimination, and public convenience must be maintained. As intelligent approval involves handling personal information, public data, and government data, it must comply with regulations in the *Personal Information Protection Law*, *Data Security Law*, *Open Government Information Regulation*, and *Regulation on Government Data Sharing*. The construction of the index should consider confidentiality obligations in information processing of administrative organs, decision-making refusal right of individuals, public data security, and government transparency. At the level of rules, State Council departments have issued rules to optimize government services, such as *Implementation Opinions on Continuously Promoting Government Service Reform and Deepening “Efficient Completion of One Matter”* by the Ministry of Housing and Urban-Rural Development, which mentions considerations for intelligent approval like home loans and public rental housing applications. Other regulatory documents like *Notice on the Construction Guidelines for the Mobile Terminal of the National Integrated Government Service Platform* and *Chongqing Municipal Government Affairs Service “One Network Access” Management Measures,* and other implementation rules and working methods provide more detailed design basis.

#### 3.2.3 Real-world conditions supply practical models.

**Fig 5:**
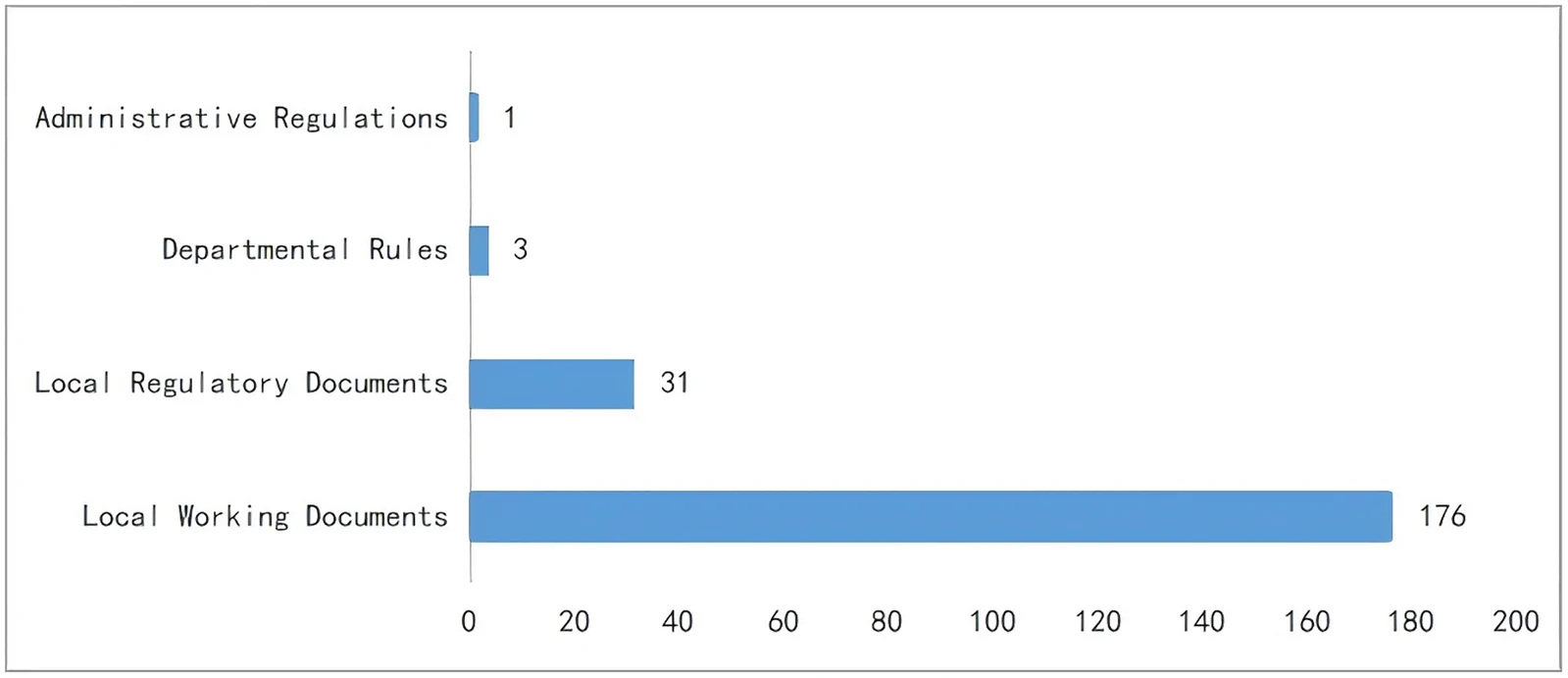
Number of documents related to the quality of administrative law enforcement (currently effective) (Data from “Peking University Law Database “, retrieved on 2026.04.28).

On one hand, extensive policy documents on law enforcement quality guide the improvement of intelligent approval. Administrative law enforcement, including intelligent approval, serves as a crucial tool for government organs to fulfill statutory functions and manage social affairs, and is a key link in legal implementation. The quality of administrative law enforcement directly impacts the effectiveness of building a digital rule-of-law government and the protection of citizens’ rights and interests. At the central level, the General Office of the State Council led the release of *The Three-Year Action Plan for Improving the Quality of Administrative Law Enforcement (2023–2025)*(hereinafter referred to as the *Quality of Administrative Law Enforcement*), setting primary objectives and tasks for quality improvement across dimensions such as administrative staff competency, law enforcement standards, working systems, supervision and guarantee. At the local level, administrative organs of provinces and cities subsequently issued local regulatory documents to enhance their law enforcement quality, responding to central guidance through specific implementation plans or ensuring ideological alignment. Fig 5 shows the number of documents across different legal hierarchies pertaining to law enforcement quality. The design and selection of intelligent approval quality evaluation index can selectively absorb and expand from these aforementioned law enforcement quality-related documents.

On the other hand, diverse approaches to law enforcement evaluation have established reference points for building an intelligent approval quality evaluation indicator system. In recent years, administrative organs and other organizations have implemented three main pathways: First, performance-based evaluations such as the “Government Department Overall Expenditure Performance Evaluation indicator system” and “Government Investment Fund Performance Evaluation indicator system” led by local government; Second, evaluations of government digitalization levels, exemplified by the *China Digital Government Development Index Report* jointly published by Tsinghua University’s Institute for Digital Government and Governance and the Center on Data and Governance of the School of Social Sciences; Third, law enforcement evaluation in the rule of law system, including Renmin University of China’s series of achievements *China Legal Development Report: Theories, Methods, and Practices of Legal System Evaluation* and Chinese Academy of Social Sciences’ *Blue Book on Rule of Law: Annual Report on Rule of Law in China*. As the connotations of the above evaluation topics overlap with intelligent approval, so quality evaluation can incorporate best practices from existing evaluations, drawing on both their macro-level architecture-such as evaluation principles, applications and subjects- and their concrete indicator design.

### 3.3 Indicators design and specific materials sources

#### 3.3.1 The theoretical framework and principles of design.

The design of evaluation indicators is not arbitrary but must adhere to relevant standards and principles, while fulfilling the ethical purpose of evaluation to enhance intelligent approval quality. Overall, the design of all indicators should, to the greatest extent possible, reflect the intrinsic character of intelligent approval systems in exercising power control and safeguarding civil rights. And they must conform to the legality defined by legal adjustments, be operable in practice, and achieve a harmonious unity of these aspects. In other words, the design should follow the coherent and systematic theoretical framework of “Value-Institution-Practice.”

The design of this indicator system must adhere to three guiding principles: First, the principle of integrating qualitative and quantitative methods. Qualitative indicators represent the values pursuit and aspirational goals of intelligent approval, such as “administrative transparency.” In practice, qualitative indicators often require quantification to enhance operational feasibility. For example, “administrative transparency” could be measured through metrics like “proactive algorithm disclosure by administrative organs” and “algorithm explanations.” Second, the principle of integrating subjective and objective methods. Since intelligent approval aims for positive social effects, it should incorporate subjective evaluation from counterparts regarding approval procedures and results. Objective indicators refer to those that are not subject to value judgment and can be evaluated only by their external performance, such as whether there is a regulatory team. Third, the principle of integrating universality with particularity. While intelligent approval shares common characteristics of universality and regularity, in view of the different development levels of various provinces and cities and the diversity of approval items, the intelligent approval of different localities and differentiated application conditions shows its own unique evaluation indicators [44].

What needs to be additionally emphasized is that, during the design phase of the indicator evaluation system, the aforementioned theoretical framework and guiding principles also possess generalizability. Their generalizability does not derive from the specific content of the indicators, but from the fact that they abstractly address the fundamental propositions and common contradictions that modern rule-of-law countries face when improving the quality of intelligent approval. Within the design theoretical framework, “value” responds to “what is the purpose of intelligent approval”; “institution” responds to “how intelligent approval is normatively constrained”; and “practice” responds to “how intelligent approval operates.” The three guiding principles provide a rigorous and scientific underlying design logic for the indicator evaluation system. Constrained by differences in administrative systems, cultural backgrounds, and other factors across countries and regions, this study can only take common problems as its orientation and strives to construct an indicator evaluation system for the quality of intelligent approval that is applicable to most modern countries. This evaluation system is suitable for both partial intelligent approval and fully intelligent approval. Scholars or administrative authorities in other countries or regions may, based on the design theoretical framework and guiding principles proposed in this study, make localized adjustments to individual indicators according to their actual circumstances, thereby forming an indicator evaluation system that meets the practical needs of their local contexts. They can complete the evaluation tasks adapted to local situations without altering the core design logic or the overall structure.

#### 3.3.2 Indicators design at all levels and corresponding materials sources.

Guided by the spirit of administrative rule of law in safeguarding civil rights and the governance context of digital government services, first-level indicators are established through the Delphi Method into four dimensions of quality: legality, rationality, satisfaction, and supervision. This study of the Delphi Method conducted two rounds of 20 experts consultations via phone, WeChat, and in-person questionnaires in April 2025.

This study strictly implemented anonymization procedures: First, all experts received questionnaires independently; the experts were unaware of each other’s identities, and no group discussions were organized. Second, after the questionnaires were collected, the researchers assigned a one-time code to each questionnaire. Third, when feeding back results to the experts, only the measures of central tendency and dispersion were reported; individual expert opinions were not presented, and no information that could identify the experts was attached. These measures ensured the anonymity requirements of the Delphi method, guaranteed that each expert’s independent judgment was free from interference by others, avoided conformity pressure, and enhanced the credibility of the research findings.

Meanwhile, this study also employed various measures to control potential biases in the Delphi method: First, the selection of experts covered different academic and practical backgrounds to control for selection bias. Second, before the study, an informed consent form was clearly provided to the experts to control for voluntary participation bias. Third, neutral language was used in the questionnaire, and experts were allowed to express open-ended opinions to control for question framing bias and researcher subjective intervention. Fourth, the anonymization procedures described above were used to control for conformity bias.

The process of this Delphi study is outlined as follows: First, establish an expert panel. In this study, the criteria for expert selection comprised both formal and substantive aspects. The formal criterion is the composition of the expert panel. Given that intelligent approval is both theoretically and practically oriented, the invited experts followed the principle of selecting interdisciplinary talents and a “scholar + practitioner” personnel structure: 2 judicial personnel (1 court official, 1 prosecutor); academics (4 law scholars, 2 management scholars, 2 sociology scholars, 2 technical experts); 3 administrative staff; and 5 legal practitioners (3 lawyers, 2 legal aid center workers). The substantive criterion is the expert’s authority, which is assessed based on four dimensions: practical experience, theoretical knowledge, awareness of peer work, and intuition. And the Expert Authority Coefficient (Cr) was 0.88 (Cr > 0.8 indicates that experts have high authority in this field and the consulting results are reliable).

Second, the first round of expert correspondence was conducted. This round was used to preliminarily determine or screen the first-level indicators for the intelligent approval quality evaluation. Compared with second-level and third-level indicators, first-level indicators are more abstract; that is, they should be established from the value dimension of intelligent approval quality. Intelligent approval is essentially a type of specific administrative act. Therefore, the formulation of first-level indicators for “intelligent approval” should align with the value pursuits of public administration and administrative law. Additionally, due to the technical nature of intelligent approval, its technical objectives should also be reflected. Hence, first-level indicators could be extracted from the theoretical foundations of relevant disciplines, legal documents, and technological applications. Although the connotation of “quality” in the context of public services has not yet been clearly defined, indicators can be derived from scholarly research explorations and relevant policy documents. Based on this, the initial set of first-level indicators for the first round of correspondence was defined as: legality, rationality, efficiency, security, satisfaction, and supervision. Questionnaires were designed accordingly to solicit experts’ opinions on these preset indicators. Experts were asked to provide comments on whether to retain, delete, or supplement each indicator, and to assign corresponding importance scores. The results indicated that the full-score frequencies for legality, rationality, satisfaction, and supervision were all above 0.6. Experts unanimously expressed strong approval of these four first-level indicators, believing that they adequately reflect the fundamental principles of lawful administration and rational administration in administrative law, and respond to the basic requirements of public perception and accountability relief in public administration and public services. In contrast, the full-score frequencies for efficiency and security were both below 0.4. Experts consider them unsuitable as primary indicators for the following reasons: they can be incorporated into other first-level indicators, their tool properties are overly pronounced, or they focus excessively on operational details.

Subsequently, the second round of correspondence was conducted. This round was intended to finalize the first-level indicators. After synthesizing the expert opinions, the experts once again expressed strong approval for using legality, rationality, satisfaction, and supervision as the first-level indicators for intelligent approval quality evaluation. They believed that these four indicators could effectively form a full-cycle evaluation framework characterized by “lawfulness before the approval, appropriateness during the approval, satisfaction after the approval, and accountability throughout the process.”

Moreover, the effective recovery rate of both rounds of questionnaires was 100%. The Kendall’s Coefficient of Concordance (Kendall’s W) was 0.350 and 0.475 respectively (Kendall’s W > 0.3 indicates that the overall consistency is valid). The Coefficient of Variation (CV) of each indicator in the first round was lower than 0.25, the CV in the second round was lower than 0.15 (CV < 0.25 indicates that the consensus of experts on a single indicator is good, and CV < 0.15 indicates that the consensus is excellent). Therefore, the correspondence could be terminated.

The corresponding second-level and third-level indicators are progressively expanded based on value guidance from the new public service theory, institutional norms in laws and policy documents, literature research, and case studies. Table 5 shows the indicators at different levels.

**Table 5:** Quality evaluation indicator system of intelligent approval.

First-level indicators	Second-level indicators	Third-level indicators
Legality	Approval according to law	Approval matters legally established Approval organs legally empowered
	Legitimate review	Prior review of approval documents Recordation review of approval documents
Rationality	Neutrality in approval	Manual approval as a fallback Setting and implementation of the green channel Specific modes of approval system Evaluate jointly the algorithm design
	Transparency in approval	Disclosure of algorithm operational logic Explain justification for non-disclosure Protect the privacy of counterpart Explain the mechanism of algorithm operation
	Fairness in approval	Restrict technical participation by negative lists Regulate the degree of technical participation by multiple standards Setting and operation of regulatory algorithm Establishment and work of regulatory departments or groups
	Participation in approval	Counterpart’s free choice or rejection of automated approval Introduce notification, statement, and defense procedures universally Access to exercise hearing right of counterpart Explain the approval system and equipment usage
Satisfaction	Outcome satisfaction	High accuracy rate of approval outcome Acceptability of approval decisions Continuous optimization of approval cycle Prompt and timely delivery of decisions
	Experience satisfaction	Reasonable setup of approval procedures Comfortable and user-friendly system interface “One Visit at Most” for services Adequate service by administrative staff
Supervision	Internal supervision	Effective hierarchical supervision within administrative organs Effective audit supervision by auditing organs Inspection of approval activities by approval organs Proper handling of administrative reconsideration
	External supervision	Implement supervision opinions from the Communist Party Implement supervision opinions from the People’s Congress Cooperate with supervision work of oversight authorities Cooperate with supervision work of judicial organs Open channels for public supervision Effective acceptance of public supervision

The fundamental dimension is “Legality.” The rule of law stands as a significant feature of modern states, particularly in China, where the spirit of rule of law is actively promoted and practiced. In this context, the legality of administrative actions has become the fundamental benchmark for evaluating law enforcement quality. This legality manifests in law-based administration through two key aspects: First, the setting of intelligent approval matters must comply with both positive enumeration and reverse limitation of laws, following the provisions of legislative hierarchy. And approval organs are strictly required to exercise their powers within legally defined jurisdictions, that is, “Approval according to law [45].” Second, to substantively ensure approval legality, documents governing approval processes undergo pre-issuance review before publication and post-issuance recordation review-a mechanism termed “Legitimate review.” The third-level indicators comprise “Approval matters legally established [46],” “Approval organs legally empowered [47],” “Prior review of approval documents [48]” and “Recordation review of approval documents [49].”

The Core dimension is “Rationality.” Law enforcement rationality is intrinsically linked to the protection of administrative counterparts’ rights, with the rationality of administrative acts serving as the core metric for evaluating enforcement quality. The reasonable administration in the *Administrative License Law* is embodied through principles of non-discrimination (neutrality), transparency, fairness, public convenience, as well as the principle of participation reflected in provisions regarding procedural rights. Given that intelligent approval inherently facilitates operational efficiency and cost reduction, the public convenience principle has been largely fulfilled through intelligent approval. Consequently, secondary indicators focus on “Neutrality in approval,” “Transparency in approval,” “Fairness in approval,” and “Participation in approval.” To achieve the above secondary indicators, in general: mitigating technical biases, enhancing algorithmic transparency, constraining algorithmic discretion, and implementing human in the loop. Specifically, the corresponding third-level indicators are “Manual approval as a fallback [50],” “Setting and implementation of the green channel (e.g., volunteer assistance for proxy services),” “Specific modes of approval system (e.g., senior citizen mode) [51],” “Evaluate jointly the algorithm design (establishing an evaluation team composed of administrative supervision, expert guidance, and technical design to conduct neutral assessments and large-scale testing of approval algorithms) [52];” “Disclosure of algorithm operational logic [53],” “(algorithms involving data security and state secrets should not be disclosed, but should) Explain justification for non-disclosure [54],” “Protect the privacy of counterpart [55],” “Explain the mechanism of algorithm operation [56];” “Restrict technical participation by negative lists (e.g., intelligent approval inapplicable to approval matters with vague conditions, complex discretion factors, conflicting interests, or emphasis on rationality judgment) [57],” “Regulate the degree of technical participation by multiple standards (e.g., difficulty of approval conditions, scope of discretion, impact on affected counterpart’s rights) [58],” “Setting and operation of regulatory algorithm,” “Establishment and work of regulatory departments or groups [59];” “Counterpart’s free choice or rejection of intelligent approval [60],” “Introduce notification, statement, and defense procedures universally [61],” “Access to exercise the right to a hearing of counterpart [62],” “Explain the approval system and equipment usage (e.g., technical standards, selection processes, accuracy rates) [63].”

The key dimension is “Satisfaction.” The service-oriented government adheres to a people-first approach, focusing on the positive impact of governance on society and public feedback regarding governmental performance. Satisfaction has become the key service indicator for evaluating law enforcement quality. Regarding satisfaction with intelligent approval, it is influenced by two factors: approval outcomes and user experience, with secondary indicators being “Outcome satisfaction” and “Experience satisfaction [64].” Tertiary indicators can be expanded into: “High accuracy rate of approval outcome (for disputed cases between counterparts and administrative organs, expert verification should be introduced to validate approval outcomes),” “Acceptability of approval decisions,” “Continuous optimization of approval cycle,” “Prompt and timely delivery of decisions;” “Reasonable setup of approval procedures,” “Comfortable and user-friendly system interface (such as prioritized interface design, categorized layouts, and AI-guided assistance),” “One Visit at Most for services” and “Adequate service by administrative staff [65].”

The accountability dimension is “Supervision.” The accountability of administrative organs and their personnel serves as the fundamental safeguard for upholding law enforcement justice [66]. A robust supervision framework has become accountability indicators to measure the quality of law enforcement. Supervision of intelligent approval is categorized into “Internal supervision” and “External supervision,” with three-tiered indicators implemented sequentially, including “Self-supervision by approval authorities [67],” “Proper handling of administrative reconsideration [68];” “Implement supervision opinions from the Communist Party [69],” “Implement supervision opinions from the People’s Congress [70],” “Cooperate with supervision work of oversight authorities [71],” “Cooperate with supervision work of judicial organs [72],” “Open channels for public supervision” and “Effective acceptance of public supervision (broadly defined to include applicants, media outlets, online self-media, and other legally authorized groups) [73].”

The framework and specific indicators of the intelligent approval quality evaluation system not only strictly adhere to the design philosophy of “value-institution-practice”, but also demonstrate practical validation of previous feasibility analysis and rule of law responses to real-world challenges in intelligent approval. This demonstrates that the system not only achieves theoretical completeness but also possesses practical feasibility.

## 4. Applying of intelligent approval quality evaluation indicator system and results analysis

**Fig 6:**
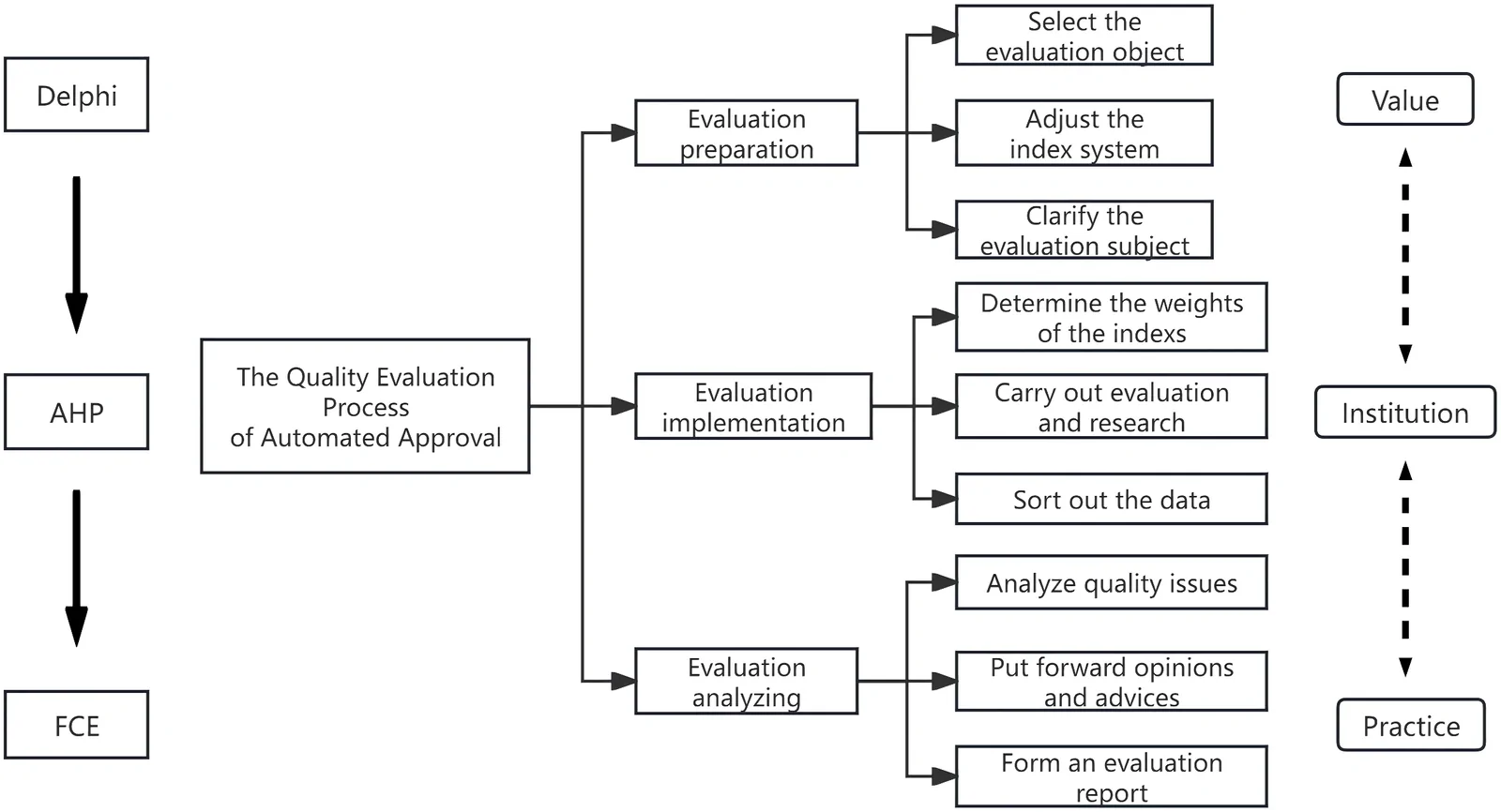
Intelligent approval quality evaluation process.

The practice of the evaluation indicator system follows a logical sequence of three phases, including preparation, implementation and analysis, with the “Value-Institution-Practice” philosophy implemented in each stage, which are shown in Fig 6. This approach ensures comprehensive evaluation under administrative values, legal norms, and empirical verification. During the preparation phase, special attention should be given to adjusting indicators according to the specific circumstances of the evaluation subject to maintain local relevance. In the implementation phase, this study proposes using the Analytic Hierarchy Process (AHP) for determining indicator weights, while adopting the Fuzzy Comprehensive Evaluation method (FCE) to evaluate. The analysis phase focuses on clarifying issues and proposing targeted solutions based on fuzzy evaluation results.

### 4.1 Preparation

The evaluation object is selected as intelligent business license approval in Taiyuan. The selection rationale includes: First, Taiyuan is a provincial capital, also a key central city and industrial hub in China with a permanent population exceeding 5 million and a 2024 GDP surpassing 500 billion yuan. Its early-stage digital government development positions it as one of China’s exemplary cities for digital governance. Second, the intelligent business license approval demonstrates typical representation, having been reported on the central government official website. This intelligent approval service covers various business license procedures including incorporation, amendment registration, cancellation, and renewal, being handled through either partially intelligent or fully intelligent approval. Third, the implementation status and prospects remain positive, with continuous improvements since its launch in 2020. Fourth, the approval has broad coverage, with corresponding intelligent facilities available at all government service centers in the city. Through communication with relevant organs and actual participation in this approval service, no adjustments were required to the original evaluation framework. Furthermore, given that certain indicators within the intelligent quality evaluation involve strong disciplinary expertise, the evaluation subjects are divided into two panels: an expert panel and a public panel, in order to conduct targeted evaluation for different types of indicators.

### 4.2 Implementation of determining the indicators weights by AHP

Firstly, This study recommends that the selection of experts for the AHP adhere to the same personnel consistency as that for the Delphi method. Maintaining consistency in the expert panel across both methods helps ensure logical coherence from “indicator screening” to “weight assignment,” avoids comprehension biases caused by differences in expert backgrounds, and thereby enhances the reliability of the indicator weights. Therefore, the 20 experts who are consistent with the Delphi experts were invited to compare the elements of each level of indicators according to the scale from 1 to 9.

Regarding the potential subjectivity issues in pairwise comparisons, this study adopted the following measures to control them. (1) Pre-training was provided to the experts to reduce subjective comprehension errors caused by unfamiliarity with AHP. The 20 experts were given a detailed explanation of the discrimination criteria and filling rules of the 1–9 scale method in AHP, along with illustrative examples. Before formal pairwise comparisons, each expert was required to complete a trial matrix unrelated to this study, and confirmed the accuracy of completion. (2) Experts were asked to make relative judgments for indicators at each level one by one, avoiding the high-load bias that could arise from completing all comparisons at once, thereby reducing the risk of subjective logical confusion due to fatigue. (3) Consistency tests were performed on the collected judgment matrices. For individual matrices with a Consistency Ratio (CR) > 0.1, the specific comparisons that violated the consistency rule were fed back to the respective experts. After revision, all matrices achieved a CR < 0.1. This feedback-revision approach improved data quality, avoiding sample loss and the potential inflation of subjectivity that would result from direct elimination.

Secondly, obtaining the relative weight of each indicator according to the above calculation steps, the calculation results are summarized in Table 6.

**Table 6:** Weights of quality evaluation indicators for intelligent approval.

First-level Indicators	Relative Weight	Second-level Indicators	Relative Weight	Third-level Indicators	Relative Weight
Legality A	0.2509	Approval according to law A1	0.6241	Approval matters legally established A11	0.5156
				Approval organs legally empowered A12	0.4844
		Legitimate review A2	0.3759	Prior review of approval documents A21	0.6605
				Recordation review of approval documents A22	0.3395
Rationality B	0.3451	Neutrality in approval B1	0.1822	Manual approval as a fallback B11	0.3649
				Setting and implementation of the green channel B12	0.3381
				Specific modes of approval system B13	0.1769
				Evaluate jointly the algorithm design B14	0.1201
		Transparency in approval B2	0.2595	Disclosure of algorithm operational logic B21	0.3181
				Explain justification for non-disclosure B22	0.1593
				Protect the privacy of counterpart B23	0.2524
				Explain the mechanism of algorithm operation B24	0.2702
		Fairness in approval B3	0.3155	Restrict technical participation by negative lists B31	0.2082
				Regulate the degree of technical participation by multiple standards B32	0.3830
				Setting and operation of regulatory algorithm B33	0.1371
				Establishment and work of regulatory departments or groups B34	0.2716
		Participation in approval B4	0.2427	Counterpart’s free choice or rejection of intelligent approval B41	0.3133
				Introduce notification, statement, and defense procedures universally B42	0.2755
				Access to exercise the right to a hearing of counterpart B43	0.2344
				Explain the approval system and equipment usage B44	0.1767
Satisfaction C	0.2529	Outcome satisfaction C1	0.7678	High accuracy rate of approval outcome C11	0.4372
				Acceptability of approval decisions C12	0.2328
				Continuous optimization of approval cycle C13	0.1065
				Prompt and timely delivery of decisions C14	0.2235
		Experience satisfaction C2	0.2322	Reasonable setup of approval procedures C21	0.2165
				Comfortable and user-friendly system interface C22	0.2516
				“One Visit at Most” for services C23	0.2751
				Adequate service by administrative staff C24	0.2568
Supervision D	0.1511	Internal supervision D1	0.4485	Effective hierarchical supervision within administrative organs D11	0.2071
				Effective audit supervision by auditing organs D12	0.1316
				Inspection of approval activities by approval organs D13	0.3429
				Proper handling of administrative reconsideration D14	0.3184
		External supervision D2	0.5515	Implement supervision opinions from the Communist Party D21	0.1744
				Implement supervision opinions from the People’s Congress D22	0.1621
				Cooperate with supervision work of oversight authorities D23	0.1551
				Cooperate with supervision work of judicial organs D24	0.1558
				Open channels for public supervision D25	0.1418
				Effective acceptance of public supervision D26	0.2108

Finally, testing the consistency according to the above calculation steps. After calculating, all the CR values are less than 0.1, which shows that all judgment matrices are considered to have satisfactory consistency and all relative weights are acceptable. And the results are summarized in the following Table 7.

**Table 7:** Summary of consistency test results.

Judgment Matrix	λmax	CI	CR
A-D	4.0309	0.0103	0.0116 < 0.1
A1-A2	2	0	0 < 0.1
B1-B4	4.0148	0.0049	0.0055 < 0.1
C1-C2	1.9999	0	0 < 0.1
D1-D2	2	0	0 < 0.1
A11-A12	2	0	0 < 0.1
A21-A22	1.9999	0	0 < 0.1
B11-B14	4.0456	0.0152	0.0171 < 0.1
B21-B24	4.0403	0.0134	0.0151 < 0.1
B31-B34	4.0357	0.0119	0.0134 < 0.1
B41-B44	4.0093	0.0031	0.0035 < 0.1
C11-C12	4.0702	0.0234	0.0263 < 0.1
C21-C22	4.0015	0.0005	0.0006 < 0.1
D11-D12	4.0557	0.0186	0.0209 < 0.1
D21-D22	6.0169	0.0034	0.0027 < 0.1

In addition, to ensure the robustness of the findings of this study, a weight perturbation test was conducted for sensitivity analysis. Perturbation tests of ±10% were performed on first-level indicator B, which had the largest relative weight, and on second-level indicator C1, which had the largest composite weight. The results are summarized in Tables 8 and 9.

**Table 8:** Results of weight perturbation test for first-level indicator B (top 10 shown).

Third-level indicator code	Original composite Weight	Weight after +10% perturbation	Weight after −10% perturbation	Original Rank	Rank after + 10% perturbation	Rank after −10% perturbation
C11	0.08493	0.08044	0.08941	1	1	1
A11	0.08074	0.07648	0.08499	2	2	2
A12	0.07586	0.07186	0.07986	3	3	3
B32	0.04171	0.04588	0.03754	6	4	8
C12	0.04521	0.04283	0.04759	4	5	4
C14	0.0434	0.04111	0.04569	5	6	5
B34	0.02958	0.03254	0.02662	7	7	9
B21	0.0285	0.03135	0.02565	8	8	10
C13	0.02069	0.0196	0.02178	9	11	7
B33	0.01493	0.01642	0.01344	12	10	13

**Table 9:** Results of weight perturbation test for first-level indicator C1 (top 10 shown).

Third-level indicator code	Original composite Weight	Weight after +10% perturbation	Weight after −10% perturbation	Original Rank	Rank after + 10% perturbation	Rank after −10% perturbation
C11	0.08493	0.09342	0.07657	1	1	1
A11	0.08074	0.08074	0.08074	2	2	2
A12	0.07586	0.07586	0.07586	3	3	3
C12	0.04521	0.04973	0.04068	4	4	5
C14	0.0434	0.04774	0.03907	5	5	6
B32	0.04171	0.04171	0.04171	6	6	4
B34	0.02958	0.02958	0.02958	7	7	7
B21	0.0285	0.0285	0.0285	8	8	8
C13	0.02069	0.02276	0.01862	9	9	9
B33	0.01493	0.01493	0.01493	10	10	10

The perturbation results for first-level indicator B showed that the rankings of the top three indicators (C11, A11, A12) remained completely unchanged, and the magnitude of weight change was consistent with the direction of perturbation. Specifically, under the + 10% perturbation: the original sixth-ranked indicator B32 rose to fourth place, while the original fourth- and fifth-ranked indicators C12 and C14 each moved down one position but remained within the top six. Under the −10% perturbation: the original fourth- and fifth-ranked indicators C12 and C14 stayed in the top five; the original ninth-ranked indicator C13 rose to seventh place; and B32 fell to eighth place. After calculation, the Spearman rank correlation coefficients (ρ) were 0.97(ρ ≥ 0.9 indicates very high robustness, with almost no substantial change in ranking, and the core conclusions are highly reliable.) and p < 0.001 for the + 10% perturbation. For the −10% perturbation, ρ were 0.96 and p < 0.001.

The perturbation results for second-level indicator C1 showed that the rankings of the top three indicators (C11, A11, A12) remained completely unchanged, and the magnitude of weight change was consistent with the direction of perturbation. Specifically, under the + 10% perturbation: the rankings remained unchanged, although the weights of some individual indicators increased. Under the −10% perturbation: the weights of C1’s subordinate indicators decreased, causing C12 and C14 to fall from fourth and fifth to fifth and sixth place respectively, while B32 rose to fourth place. Nevertheless, C13 remained in ninth place and B33 remained in tenth place. For the + 10% perturbation, ρ were 0.98 and p < 0.001. For the −10% perturbation, ρ were 0.96 and p < 0.001.

The results of this weight perturbation test indicate that when the weights of first-level and second-level indicators fluctuate within a range of ±10%, the rankings of the composite weights of the corresponding third-level indicators exhibit good statistical robustness. Therefore, the AHP weight results of this study are reliable and credible, and the core conclusions are not affected by weight errors within a reasonable range.

### 4.3 Implementation of carrying out evaluation and calculating scores by FCE

First, given that some indicators have high professional barriers, while others are closely related to the participation experience of administrative counterparts, the selection of evaluators should follow the principle of combining experts with the general public. This ensures that the evaluation results reflect professionalism, rigor, and authority, while also fully and broadly capturing the genuine perceptions of administrative counterparts regarding the quality of intelligent approval. Therefore, 10 experts and 100 members of the public in Taiyuan were invited to evaluate the quality of intelligent approval. The expert panel combines scholars and practitioners: 3 legal scholars, 1 management scholar, 1 sociology scholar, 2 administrative personnel, 1 court staff member, 1 procuratorate staff member, and 1 lawyer. The public panel was selected through sampling survey method. T City administers 10 districts and counties, with each district selecting 10 commercial entities that had previously used the intelligent business license approval system in Taiyuan. The expert group is primarily responsible for evaluating indicators that require strong professional expertise, such as most indicators under approval legality and approval supervision. The public group is primarily responsible for evaluating indicators that are experience- and perception-oriented, such as most indicators under approval rationality and approval satisfaction.

This study adopts an evaluation result integration mechanism of “stratified evaluation + overall aggregation” for the expert group and the public group. Specifically, at the third-level indicator level, since the expert group and the public group are responsible for different third-level indicators, the membership degree and score of each third-level indicator are calculated separately. At the second-level and first-level indicator levels, because membership degrees can standardize the evaluations of both the expert group and the public group, the evaluation results of the third-level indicators can be directly synthesized layer by layer according to their weights, ultimately yielding the second-level indicators, first-level indicators, and the comprehensive evaluation score for intelligent approval quality. There is no need to assign additional integration weights to the two groups separately, thereby avoiding subjective bias caused by secondary weighting.

Second, tabulating the evaluation data and calculating the membership degree of three-level indicators (the proportion of evaluators who assign a particular grade to the i-th factor relative to the total number of participants in that evaluation).The results are shown in Table 10:

**Table 10:** Membership degree of three-level indicators of business license intelligent approval quality.

Third-level indicators	Evaluation subject	The membership degree				
		Excellent	Good	Average	Poor	Very Poor
Approval matters legally established A11	Experts	1.00	0.00	0.00	0.00	0.00
Approval organs legally empowered A12	Experts	1.00	0.00	0.00	0.00	0.00
Prior review of approval documents A21	Experts	0.90	0.10	0.00	0.00	0.00
Recordation review of approval documents A22	Experts	0.80	0.20	0.00	0.00	0.00
Manual approval as a fallback B11	The public	0.76	0.12	0.08	0.03	0.01
Setting and implementation of the green channel B12	The public	0.32	0.36	0.21	0.05	0.06
Specific modes of approval system B13	The public	0.15	0.29	0.44	0.09	0.03
Evaluate jointly the algorithm design B14	Experts	0.50	0.20	0.20	0.10	0.00
Disclosure of algorithm operational logic B21	The public	0.00	0.00	0.00	0.82	0.18
Explain justification for non-disclosure B22	The public	0.00	0.00	0.00	0.73	0.27
Protect the privacy of counterpart B23	The public	0.91	0.06	0.03	0.00	0.00
Explain the mechanism of algorithm operation B24	The public	0.00	0.00	0.00	0.66	0.34
Restrict technical participation by negative lists B31	Experts	0.33	0.40	0.20	0.00	0.10
Regulate the degree of technical participation through multiple standards B32	Experts	0.30	0.50	0.10	0.10	0.00
Setting and operation of regulatory algorithm B33	Experts	0.60	0.33	0.10	0.00	0.00
Establishment and work of regulatory departments or groups B34	Experts	0.40	0.20	0.20	0.10	0.00
Counterpart’s free choice or rejection of intelligent approval B41	The public	0.93	0.03	0.04	0.00	0.00
Introduce notification, statement, and defense procedures universally B42	The public	0.36	0.32	0.30	0.02	0.00
Access to exercise the right to a hearing of counterpart B43	The public	0.08	0.05	0.67	0.06	0.04
Explain the approval system and equipment usage B44	The public	0.94	0.05	0.01	0.00	0.00
High accuracy rate of approval outcome C11	The public	0.90	0.04	0.04	0.02	0.00
Acceptability of approval decisions C12	The public	0.83	0.09	0.03	0.04	0.01
Continuous optimization of approval cycle C13	The public	0.73	0.11	0.12	0.02	0.02
Prompt and timely delivery of decisions C14	The public	0.98	0.02	0.00	0.00	0.00
Reasonable setup of approval procedures C21	The public	0.52	0.38	0.10	0.00	0.00
Comfortable and user-friendly system interface C22	The public	0.77	0.21	0.01	0.01	0.00
“One Visit at Most” for services C23	The public	0.34	0.37	0.15	0.05	0.09
Adequate service by administrative staff C24	The public	0.24	0.16	0.43	0.07	0.10
Effective hierarchical supervision within administrative organs D11	Experts	0.80	0.00	0.20	0.00	0.00
Effective audit supervision by auditing organs D12	Experts	0.80	0.20	0.00	0.00	0.00
Inspection of approval activities by approval organs D13	Experts	0.60	0.10	0.00	0.10	0.20
Proper handling of administrative reconsideration D14	The public	0.33	0.31	0.35	0.01	0.00
Implement supervision opinions from the Communist Party D21	Experts	0.90	0.00	0.10	0.00	0.00
Implement supervision opinions from the People’s Congress D22	Experts	0.80	0.10	0.10	0.00	0.00
Cooperate with supervision work of oversight authorities D23	Experts	0.70	0.10	0.20	0.00	0.00
Cooperate with supervision work of judicial organs D24	Experts	0.60	0.20	0.10	0.10	0.00
Open channels for public supervision D25	The public	0.16	0.26	0.57	0.06	0.05
Effective acceptance of public supervision D26	The public	0.11	0.21	0.33	0.29	0.06

Last, calculating the membership degree of second-level and third-level indicators layer and the fuzzy evaluation scores according to the above calculation steps. Take category A indicators as an example:

(1) According to the membership degree in Table 10, fuzzy evaluation is carried out on the third-level indicators A11 and A12 according to their membership degree *Z*_*A1*1_ and *Z*_*A12*_ respectively, and their evaluation scores are as follows:

$$\begin{array}{c} Y_{A\text{11}}=Z_{A\text{11}}\times V=[\begin{array}{ccccc} \text{1}.\text{00} & \text{0}.\text{00} & \text{0}.\text{00} & \text{0}.\text{00} & \text{0}.\text{00} \end{array}]\times [\begin{array}{c} \text{90}\\ \text{70}\\ \text{50}\\ \text{30}\\ \text{10} \end{array}]=\text{90 }(\text{Excellent}) \end{array}$$

$$\begin{array}{c} Y_{A\text{12}}=Z_{A\text{12}}\times V=[\begin{array}{ccccc} \text{1}.\text{00} & \text{0}.\text{00} & \text{0}.\text{00} & \text{0}.\text{00} & \text{0}.\text{00} \end{array}]\times [\begin{array}{c} \text{90}\\ \text{70}\\ \text{50}\\ \text{30}\\ \text{10} \end{array}]=\text{90 }(\text{Excellent}) \end{array}$$

(2) According to the indicator weight in Table 6 and the membership degree in Table 8, fuzzy evaluation is carried out on the second-level indicator A1. Its weight set and fuzzy matrix are *W*_*A1*_ and *F*_*A1*_ respectively:

$$\begin{array}{c} W_{A\text{1}}=[\begin{array}{cc} \text{0}.\text{5156} & \text{0}.\text{4844} \end{array}] \end{array}$$

$$\begin{array}{c} F_{A\text{1}}=\left[\begin{array}{ccc} \text{1}.\text{00} & \text{0}.\text{00} & \text{0}.\text{00}\\ \text{1}.\text{00} & \text{0}.\text{00} & \text{0}.\text{00} \end{array}\;\begin{array}{cc} \text{0}.\text{00} & \text{0}.\text{00}\\ \text{0}.\text{00} & \text{0}.\text{00} \end{array}\right] \end{array}$$

Calculate the membership degree of A1:


$$\begin{array}{c} Z_{A\text{1}}=W_{A\text{1}}\times F_{A\text{1}}=[\begin{array}{ccccc} \text{1}.\text{00} & \text{0}.\text{00} & \text{0}.\text{00} & \text{0}.\text{00} & \text{0}.\text{00} \end{array}] \end{array}$$

Calculate the score of A1:


$$\begin{array}{c} Y_{A\text{1}}=Z_{A\text{1}}\times V=[\begin{array}{ccccc} \text{1}.\text{00} & \text{0}.\text{00} & \text{0}.\text{00} & \text{0}.\text{00} & \text{0}.\text{00} \end{array}]\times [\begin{array}{c} \text{90}\\ \text{70}\\ \text{50}\\ \text{30}\\ \text{10} \end{array}]=\text{90 }(\text{Excellent}) \end{array}$$

(3) According to the membership degree in Table 10, fuzzy evaluation is carried out on the third-level indicators A21 and A22 according to their membership degree *Z*_*A21*_ and *Z*_*A22*_ respectively, and their evaluation scores are as follows:

$$\begin{array}{c} Y_{A\text{21}}=Z_{A\text{21}}\times V=[\begin{array}{ccccc} \text{0}.\text{90} & \text{0}.\text{10} & \text{0}.\text{00} & \text{0}.\text{00} & \text{0}.\text{00} \end{array}]\times [\begin{array}{c} \text{90}\\ \text{70}\\ \text{50}\\ \text{30}\\ \text{10} \end{array}]=\text{88 }(\text{Excellent}) \end{array}$$

$$\begin{array}{c} Y_{A\text{22}}=Z_{A\text{22}}\times V=[\begin{array}{ccccc} \text{0}.\text{80} & \text{0}.\text{20} & \text{0}.\text{00} & \text{0}.\text{00} & \text{0}.\text{00} \end{array}]\times [\begin{array}{c} \text{90}\\ \text{70}\\ \text{50}\\ \text{30}\\ \text{10} \end{array}]=\text{86 }(\text{Excellent}) \end{array}$$

(4) According to the indicator weight in Table 6 and the membership degree in Table 7, fuzzy evaluation is carried out on the second-level indicator A2. The weight set and fuzzy matrix are *W*_*A2*_ and *F*_*A2*_ respectively:

$$\begin{array}{c} W_{A\text{2}}=[\begin{array}{cc} \text{0}.\text{6605} & \text{0}.\text{3395} \end{array}] \end{array}$$

$$\begin{array}{c} F_{A\text{2}}=\left[\begin{array}{ccc} \text{0}.\text{90} & \text{0}.\text{10} & \text{0}.\text{00}\\ \text{0}.\text{80} & \text{0}.\text{20} & \text{0}.\text{00} \end{array}\;\begin{array}{cc} \text{0}.\text{00} & \text{0}.\text{00}\\ \text{0}.\text{00} & \text{0}.\text{00} \end{array}\right] \end{array}$$

Calculate the membership degree of A2:


$$\begin{array}{c} Z_{A\text{2}}=W_{A\text{2}}\times F_{A\text{2}}=[\begin{array}{ccccc} \text{0}.\text{86605} & \text{0}.\text{13395} & \text{0}.\text{00} & \text{0}.\text{00} & \text{0}.\text{00} \end{array}] \end{array}$$

Calculate the score for A2:


$$\begin{array}{c} Y_{A\text{2}}=Z_{A\text{2}}\times V=[\begin{array}{ccccc} \text{0}.\text{86605} & \text{0}.\text{13395} & \text{0}.\text{00} & \text{0}.\text{00} & \text{0}.\text{00} \end{array}]\times [\begin{array}{c} \text{90}\\ \text{70}\\ \text{50}\\ \text{30}\\ \text{10} \end{array}]=\text{87}.\text{321 }(\text{Excellent}) \end{array}$$

(5) According to the indicator weight in Table 6, the membership degree in Table 8, and the fuzzy evaluation results of the above second-level indicators A1 and A2, the fuzzy evaluation of the first-level indicator A is carried out, and its weight set and fuzzy matrix are *W*_*A*_ and *F*_*A*_ respectively:

$$\begin{array}{c} W_{A}=[\begin{array}{cc} \text{0}.\text{6241} & \text{0}.\text{3759} \end{array}] \end{array}$$

$$\begin{array}{c} F_{A}=\left[\begin{array}{ccc} \text{1}.\text{00} & \text{0}.\text{00} & \text{0}.\text{00}\\ \text{0}.\text{86605} & \text{0}.\text{13395} & \text{0}.\text{00} \end{array}\;\begin{array}{cc} \text{0}.\text{00} & \text{0}.\text{00}\\ \text{0}.\text{00} & \text{0}.\text{00} \end{array}\right] \end{array}$$

Calculate the membership degree of A:


$$\begin{array}{c} Z_{A}=W_{A}\times F_{A}=[\begin{array}{ccccc} \text{0}.\text{949648195} & \text{0}.\text{050351805} & \text{0}.\text{00} & \text{0}.\text{00} & \text{0}.\text{00} \end{array}] \end{array}$$

Calculate the score of A:


$$\begin{array}{c} Y_{A}=Z_{A}\times V=[\begin{array}{ccccc} \text{0}.\text{949648195} & \text{0}.\text{050351805} & \text{0}.\text{00} & \text{0}.\text{00} & \text{0}.\text{00} \end{array}]\times [\begin{array}{c} \text{90}\\ \text{70}\\ \text{50}\\ \text{30}\\ \text{10} \end{array}]=\text{88}.\text{9929639 }(\text{Excellent}) \end{array}$$

Similarly, the fuzzy evaluation of other indicators was carried out step by step and the evaluation scores were calculated. The final results are shown as follows in Table 11. For readability and maintaining a tidy table, the contents of “Grade” are abbreviated as *E*(Excellent), *G*(Good), *A*(Average), *P*(Poor), VP (Very Poor).

**Table 11:** Comprehensive fuzzy evaluation score and grade of intelligent approval quality in Taiyuan.

First-level indicators	Score	Grade	Second-level indicators	Score	Grade	Third-level indicators	Score	Grade
Legality A	88.9929639	*E*	Approval according to law A1	90	*E*	Approval matters legally established A11	90	*E*
						Approval organs legally empowered A12	90	*E*
			Legitimate review A2	87.321	*E*	Prior review of approval documents A21	88	*E*
						Recordation review of approval documents A22	86	*E*
Rationality B	63.31589401	*G*	Neutrality in approval B1	71.4152	*G*	Manual approval as a fallback B11	81.8	*E*
						Setting and implementation of the green channel B12	66.6	*G*
						Specific modes of approval system B13	58.8	***A***
						Evaluate jointly the algorithm design B14	72	*G*
			Transparency in approval B2	40.6955	***A***	Disclosure of algorithm operational logic B21	26.4	***P***
						Explain justification for non-disclosure B22	24.6	***P***
						Protect the privacy of counterpart B23	87.6	*E*
						Explain the mechanism of algorithm operation B24	23.2	***P***
			Fairness in approval B3	69.48005	*G*	Restrict technical participation by negative lists B31	68.7	*G*
						Regulate the degree of technical participation through multiple standards B32	70	*G*
						Setting and operation of regulatory algorithm B33	82.1	*E*
						Establishment and work of regulatory departments or groups B34	63	*G*
			Participation in approval B4	73.43472	*G*	Counterpart’s free choice or rejection of intelligent approval B41	87.8	*E*
						Introduce notification, statement, and defense procedures universally B42	70.4	*G*
						Access to exercise the right to a hearing of counterpart B43	46.4	***A***
						Explain the approval system and equipment usage B44	88.6	*E*
Satisfaction C	82.47496916	*E*	Outcome satisfaction C1	85.84962	*E*	High accuracy rate of approval outcome C11	86.4	*E*
						Acceptability of approval decisions C12	83.8	*E*
						Continuous optimization of approval cycle C13	80.2	*E*
						Prompt and timely delivery of decisions C14	89.6	*E*
			Experience satisfaction C2	71.31624	*G*	Reasonable setup of approval procedures C21	78.4	*G*
						Comfortable and user-friendly system interface C22	84.8	*E*
						“One Visit at Most” for services C23	66.4	*G*
						Adequate service by administrative staff C24	57.4	***A***
Supervision D	72.69620834	*G*	Internal supervision D1	72.96448	*G*	Effective hierarchical supervision within administrative organs D11	82	*E*
						Effective audit supervision by auditing organs D12	86	*E*
						Inspection of approval activities by approval organs D13	66	*G*
						Proper handling of administrative reconsideration D14	69.2	*G*
			External supervision D2	72.47804	*G*	Implement supervision opinions from the Communist Party D21	86	*E*
						Implement supervision opinions from the People’s Congress D22	84	*E*
						Cooperate with supervision work of oversight authorities D23	80	*E*
						Cooperate with supervision work of judicial organs D24	76	*G*
						Open channels for public supervision D25	63.4	*G*
						Effective acceptance of public supervision D26	50.4	***A***
Final evaluation score and evaluation grade						76.020966446099 *G*		

### 4.4 Results analysis

#### 4.4.1 Evaluation score and grade analysis.

According to the above evaluation study, the overall quality score of business license intelligent approval in T city shows about 76.02, which stands for good.

Regarding first-level and second-level indicators: The first-level indicator “Legality-A” with its secondary indicators achieve the excellent grade. The first-level indicator “Rationality-B” with its secondary indicators obtain a good grade except the average grade of “Transparency in approval-B2”. The first-level indicator “Satisfaction-C” and its secondary indicator “Outcome satisfaction-C1” meet the excellent grade, while its sub-indicator “Experience satisfaction-C2” meets the good grade. The first-level indicator “Supervision-D” and its secondary indicators all reveal the good grade.

Among third-level indicators: 19 are evaluated as excellent (accounting for 50% of total third-level indicators), which mainly concentrate on the dimension of “Legality-A” and “Outcome satisfaction-C1”. 12 are evaluated as good (accounting for approximately 31.58%), which are relatively scattered. 4 are evaluated as average (accounting for approximately 10.53%), including “Specific modes of approval system-B13”, “Access to exercise the right to a hearing of counterpart-B43”, “Adequate service by administrative staff-C24” and “Effective acceptance of public supervision-D26”. 3 are evaluated as poor (accounting for approximately 7.89%), including “Disclosure of algorithm operational logic-B21”, “Explain justification for non-disclosure-B22”, and “Explain the mechanism of algorithm operation-B24”. 0 are evaluated as very poor.

#### 4.4.2 Quality problems analysis.

According to the above empirical research and results sorting, there are potential quality problems and prominent quality problems in the intelligent approval of business license in Taiyuan.

Potential quality problems are evident in indicators rated as the grade of average, which are reflected in rationality, satisfaction, and supervision. The rationality dimension includes “Specific modes of approval system-B13” of neutrality and “Access to exercise the right to a hearing of counterpart-B43” of participation. Although the approval system does have specific modes like voice prompts, they’re not sufficient enough and sometimes ineffective, which causes the average grade of B13. And the process of intelligent approval is too fast to exercise the right of hearing, especially the fully intelligent approval, so that B43 is rated the average grade. The satisfaction dimension includes “Adequate service by administrative staff-C24” of experience satisfaction. Notwithstanding the enhanced professional caliber of the administrative personnel, there are persistent shortfalls in the clarity of directives provided. Furthermore, the current staffing levels are insufficient to handle the volume of requests. Consequently, this indicator C24 has been assigned the average grade. The supervisory dimension demonstrates “Effective acceptance of public supervision-D26” of external supervision. There are indeed dedicated complaint channels, but the feedback time was slightly longer, and some of the feedback failed to fully convince the applicant, which all contribute to D26’s average grade. In summary, these indicate that the approval system’s special modes for digitally disadvantaged groups are limited and malfunctioning, the right to a hearing conditions remain inadequate, and administrative staff’s service competence and feedback mechanisms require continuous improvement. At its core, the above potential problems are mainly due to the imperfect institutional safeguards for digitally disadvantaged groups and procedural rights. In the process of smart and service-oriented transformation of modern cities, a perspective centered on the subject is indispensable. The *2023 White Paper on Digital Competence of Chinese Citizens* points out that China’s digital economy ranks second in the world, yet the overall score of the Chinese citizens’ digital competence index is at a medium global level. This indicates a mismatch between the development of China’s digital economy and the improvement of citizens’ digital literacy; digitally disadvantaged groups remain a segment that cannot be overlooked in the promotion of intelligent government services. In fact, many regions have already begun to pay increasing attention to safeguarding these groups. For example, the European Union enacted the *Digital Service Act* in 2022, which includes provisions on accessible digital services, requiring public sector administrative services to provide adaptive solutions for digitally disadvantaged groups. Similarly, China’s Hunan Province issued the *Several Provisions on the Construction of a Barrier-Free Environment* in 2025, the first local regulation in China that systematically integrates information accessibility into smart city construction, making accessibility provision a statutory duty of public service departments. However, relevant authorities are still deficient in responding to the needs of digitally disadvantaged groups and delivering adequate services, which explains why the associated evaluation results are only average. Regarding the adaptive renewal of procedural rights in the context of technological evolution, the academic community has already provided answers by proposing the concept of “technological due process” and conducting extensive theoretical explorations. Yet current practice still lags behind theory, while also facing the test of local feasibility. As a result, the exercise of the right to a hearing remains difficult to fully implement.

The prominent quality problems are reflected in the indicators that are evaluated as the grade of poor, focusing on transparency of approval in rationality: “Disclosure of algorithm operational logic-B21”, “Explain justification for non-disclosure-B22”, and “Explain the mechanism of algorithm operation-B24”. The approval platform only provides step-by-step instructions without disclosing how approval algorithms operate, this leads to the poor grade for B21. And the administrative organs have not offered any justification to the public for its decision to unreveal the algorithm operation, which results in B22 being evaluated as poor. Additionally, since the algorithm operation is not disclosed, there is no explanation of how it works, which contributes to the poor grade for B24. That is to say, administrative organs failed to proactively disclose or explain the operational mechanisms of approval algorithms, nor did they provide justification, failing to uphold the principle of transparency in rational administration. From the perspectives of traditional administrative law and public administration, administrative openness is a fundamental principle that administrative agencies must observe when making administrative decisions, and it is also an inevitable requirement for a modern rule of law government to protect the public right to know. As the digital process accelerates, algorithms have become the vehicle for decision-making in intelligent government services, and algorithmic transparency has thus become an extension of the principle of administrative openness in the digital age. At the international level, algorithmic transparency is gradually moving from a principle to a concrete legal obligation. For example, the UK issued the *Algorithmic Transparency Recording Standard* in 2024, which aims to help public sector bodies clearly provide information about the algorithmic tools they use in decision-making processes in a standardized manner, with all records to be centrally stored on the GOV. UK website. In 2025, Amendment 119 to the *UK Data (Use and Access) Act* proposed elevating the application of the Algorithmic Transparency Recording Standard from administrative guidance to a statutory duty through legislation. Another example is the *Canada Directive on Automated Decision-Making*, which requires all federal agencies to publish explanations of their AI driven decisions. In contrast, current Chinese discussions on algorithmic transparency remain mostly at the theoretical level, and no clear legal rules have been enacted to specifically regulate algorithmic transparency in practice. This leaves administrative authorities lacking authoritative and specific normative requirements, resulting in a lag in the development of algorithmic transparency.

#### 4.4.3 Suggestions for quality improvement.

For the quality problems of intelligent approval derived from the evaluation indicator system, on the one hand, we should prevent potential quality problems to avoid them developing into significant problems; on the other hand, we had better solve the prominent quality problems to eliminate them in time.

Prevent potential quality problems by continuously optimizing intelligent approval. First, the digital infrastructure should be upgraded. Approval systems or devices should introduce diverse user-care modes such as “elder mode”, “text-to-speech”, “voice navigation”, and “page pinyin” to build a human rights platform for equal access to digital approval. Second, the right to a hearing must be strictly protected. While Chinese legislation grants parties the right to a hearing, the form of hearing has not been specified. Generally, before the approval decision is made, manual approval requires prior or in-process hearings to meet “prior notification” requirements under due process. These two types of hearing are still applicable to partially intelligent approval that meets the requirements. The procedures which administrative staff and technology may participate in should be both set up with “prior notification”. Additionally, given the instantaneous nature of fully intelligent decisions, post-event hearings should be permitted when necessary. Third, enhance administrative staff service competence. Service counters for vulnerable groups should be established with professional guides, such as volunteer teams assisting seniors with proxy services to ensure full-process support. Simultaneously, training programs should adapt to automation features, like the administrative information management system training conducted by Xinxiang City in Henan Province for city-wide staff, aiming to improve both service and technical competencies. Fourth, strengthen public feedback mechanisms. Public opinions on administrative approval serve as crucial supervisory channels. Administrative organs should cooperate with public supervision by accelerating response and continuous follow-up, incorporating these into long-term government performance evaluations.

Address prominent quality problems by enhancing algorithm transparency. The significant issue in the intelligent business license approval in Taiyuan stems from insufficient transparency of administrative algorithms, which risks creating the “algorithmic black box”. As U.S. Justice Brandeis famously stated, “Sunlight is the best disinfectant” [74], therefore governments should proactively disclose their approval algorithms to achieve “algorithmic transparency”. First, clearly defining the scope of algorithm disclosure to achieve form transparency. Three approaches can be adopted: 1) Non-disclosure baseline: Algorithms involving data security and state secrets (e.g., customs import/export approval, intellectual property approval) should remain confidential. However, governments may disclose such algorithms under the “principle of distinction” after excluding data compromising national security or sensitive information. Additionally, undisclosed algorithms require justification. 2) Relative exemption from disclosure: Personal privacy-related content should generally remain confidential, with disclosure permitted only through individual consent or public interest considerations [75]. 3) Full disclosure: Article 7 of the *Personal Information Protection Law* specifies disclosure criteria-purpose, method, and scope for personal data processing activities, indicating that administrative organs must disclose algorithmic approval items, operational mechanisms, and types of rights affected by these systems. While the *Data Security Law* does not specify disclosure parameters, government data inherently refers to digitized government information. Given that the *Open Government Information Regulation* establishes disclosure as the norm, administrative organs must fully disclose algorithmic source code and intermediate processes (including inputs and outputs). Second, transparent algorithmic explanations are essential for achieving substantive transparency-a contemporary imperative of “technological due process” in administrative acts. Notable examples include the *Algorithm Charter for Aotearoa New Zealand* mandating public explanation of algorithms to enhance transparency, and the *French Digital Republic Act* granting citizens the right to request algorithmic explanations of algorithmic administrative decisions about individuals [76]. Although China’s government information disclosure system lacks explicit explanation obligation, the principle of due process and the *Administrative License Law* require organs to provide justification explanations, and Article 48 of the *Personal Information Protection Law* explicitly grants individuals the right to request explanation of their information processing rules. As the primary subjects responsible for government information disclosure and personal data processing, administrative organs must clearly explain algorithms’ objectives, operational logic, formulas, consideration factors, and proportion, decoded laws, and associated data in a manner accessible to the public. This ensures intelligent approval processes are identifiable, understandable, and traceable, thereby enhancing transparency and knowability in administrative decision-making and predictability of outcomes [77]. Such measures help the public overcome algorithmic comprehension barriers while driving a paradigm shift from “algorithm-centered” to “human-centered” algorithm interpretation [78].

## 5. Discussion

### 5.1 Significance and highlights

This study contributes to both theoretical and empirical research on the harmonious integration of technology and society by developing a nationwide quality evaluation system for intelligent approval and conducting a case study in Taiyuan, which aims to make targeted improvements to government services and protect the rights of counterparts, thereby facilitating digital rule of law government and advancing the sustainable planning of smart cities.

The empirical analysis of evaluation demonstrates that the evaluation indicator system developed in this study can accurately identify quality issues in intelligent approval processes with practical operability. The evaluation study has received positive feedback from relevant personnel: scholars involved in the evaluation process noted that the system demonstrates innovative value, practitioners highlighted its practical significance as a quantifiable evaluation method for law enforcement, and the public acknowledged that visualized data allows citizens to tangibly perceive the progress of digital governance initiatives. The evaluation results and the above feedback show that the quality evaluation indicator system is suitable for promotion across provinces and cities to identify quality problems of intelligent approval and explore the corresponding solutions, thereby advancing government services.

The highlights of this study are mainly reflected in four aspects. First, the specificity of the research subjects. Compared with previous macro research, this study selects intelligent approval, which is more in demand, more mature technologies, greater public perceptibility of its service, and is widely practiced by the public.

Second, the compositeness and replicability of the research methods. Compared with previous theoretical studies, this study adopts a multidisciplinary approach (public administration, administrative law, sociology, etc.) and empirical research methods (Delphi, AHP, and FCE) to construct a complete and replicable indicator system for evaluating the quality of intelligent approval. It breaks through the previous qualitative analysis of specific digital administrative acts by means of quantitative research.

Third, the research content possesses substantial academic value. On the one hand, against the backdrop of smart city construction and digital government services, this study systematically identifies the core quality issues of intelligent approval from a theoretical perspective, conducts an in-depth analysis of the research results, and explores their underlying theoretical significance. At the same time, it constructs corresponding institutional frameworks at the theoretical level to address the quality problems identified through empirical research, effectively responding to hot-topic issues such as the protection of vulnerable groups under intelligent decision-making and procedural justice. On the other hand, this study innovatively proposes a “Value-Institution-Practice” theoretical framework for indicator design, filling the research gap caused by the lack of systematic theoretical support in the current quantitative research on digital administrative acts.

Fourth, the feasibility of the indicator system. The theoretical framework of the indicator design is not only geographically generalizable but can also be extended to quantitative research on other digital administrative acts. Moreover, the indicator system has been successfully implemented in practical applications, fully demonstrating its strong practicality and its ability to provide robust support for smart city planning and government service optimization.

### 5.2 Limitations and future research

While the empirical research proceeded smoothly overall, several limitations and challenges emerged. First, this evaluation study led solely by scholars results in the lack of organization, thereby impeding the smooth execution of the evaluation process. In addition, the time-consuming data collection and processing reduced efficiency. Furthermore, the absence of specific institutional guidelines for digital administrative act evaluation undermines the authority and standardization. Therefore, these shortcomings should be addressed during implementation to ensure effective practice and promotion in the future.

Firstly, establish a dedicated evaluation committee. On the one hand, the committee must operate independently from approval departments. From the perspective of administrative organization, under the traditional hierarchical organizational framework, self-supervision by administrative agencies leads to the structural dilemma of “judging one’s own case.” Moreover, administrative agencies tend to prioritize administrative efficiency and often treat the improvement of government service quality merely as one of the performance assessment goals, making it difficult to genuinely address substantive issues and implement corrective measures. Rosenbloom, a renowned American public administration scholar, emphasized that when an administrative organ performs quasi-judicial functions, the organizational design of the administration should adopt the form of an independent commission [79]. In the quality assessment of intelligent approval, evaluating whether the approval system affects administrative transparency, due process, the rights of private parties, and the rights of vulnerable groups is essentially a manifestation of quasi-judicial functions; therefore, the assessment should be carried out by a specialized independent commission. Examples include the French High Authority for Transparency in Public Life and the Japan Personal Information Protection Commission, both of which supervise public power and protect private rights in the form of independent commissions [80]. Consequently, an independent evaluation commission has both theoretical support and practical foundations.

On the other hand, since intelligent approval quality evaluation is a systematic operation requiring coordinated efforts, collaborative governance should actively guide the evaluation process. Collaborative governance originates from Synergetics in the natural sciences in the 1970s. In the field of public administration, it is an operational mechanism that brings together different actors to jointly achieve governance objectives [81]. Compared with Public-Public Collaboration, Public-Private Collaboration is better able to highlight the distinctive advantages of multiple parties in the current context of social co-governance and pluralistic interests. Specifically, the public sector acts as the initiator of collaborative governance, undertaking the key task of solving public problems and creating public value [82]. Private actors, on the other hand, primarily play roles in improving efficiency, enhancing resource utilization, and innovating methods, while also granting legitimacy and rationality to collaborative governance, making it more procedurally fair, and strengthening accountability [83]. Therefore, the evaluation committee may be led by oversight authorities with support from government legal offices, finance departments, and audit departments in terms of resources and funding, to ensure the committee’s work is well-organized, standardized, and sustainable. It should include scholars from law, science of public administration, and sociology disciplines, along with practitioners, public representatives, and technical experts. So as to ensure that its composition possesses both professional authority and diversity, fully leverages the strengths of different actors in policy understanding, academic research, technical judgment, and user perception, avoids the perspective bias that may arise from assessment by a single actor, and thereby enhances the comprehensiveness and credibility of the evaluation results.

Secondly, leveraging the precision and efficiency of digital platforms. The three empirical methods employed in this study formally take the form of electronic or paper-based correspondence and questionnaires. Given that this study serves academic and public interests, has no profit-making intent, and participants receive no academic labor compensation, the financial cost of the empirical research is primarily focused on printing supplies and transportation expenses within Taiyuan City, which remains within an acceptable range. However, if the improvement suggestion of establishing a dedicated evaluation committee in the future is followed, the relevant authorities should set up a special fund for academic labor compensation to reward research participants and enhance their motivation to engage. In terms of time cost, overall, the three empirical studies spanned approximately five months, from around April 2025 to around August 2025. Specifically: the Delphi method involved two rounds of expert correspondence. The first round, used to screen the first-level indicators, took about three weeks (including data collation). The second round, used to confirm the first-level indicators, also took about three weeks (including data collation). The AHP took about one month (including data collation). The FCE took about two months (including data collation). The overall progress aligns with the conventional research timeline for these three empirical methods and does not pose a major obstacle to the advancement of the research. After reflection, the main reason for the longer time spent on the FCE is the relative difficulty of offline collection of public assessments. In addition, data collation also requires a certain amount of time. Future work should implement targeted improvements to optimize time utilization and enhance evaluation efficiency. On the one hand, since intelligent approval primarily relies on central or provincial/municipal online government service platforms, evaluation works can be seamlessly integrated into these platforms. Therefore, for public evaluation indicators, administrative organs may promptly invite citizens to participate in online surveys either at the application completion stage or after approval results are delivered, preventing accuracy and effectiveness problems caused by prolonged processing times. Regarding experts’ evaluation indicators, organs should regularly encrypt and archive relevant materials for future reference. On the other hand, while the calculation methods involved in intelligent approval quality evaluation are not overly complex, they are more tedious. Researchers could create auxiliary calculation programs to streamline workflows, ensuring data accuracy and optimizing resource allocation for evaluators.

Finally, efforts should be intensified to advance legislation for administrative law enforcement evaluation to explore its theoretical foundations. The quality evaluation of intelligent approval involves not only management and technical considerations but also a legal dimension, with legislative frameworks serving as institutional safeguards for effective implementation. From a global law perspective, legislative practices in this field have already been established, exemplified by the *Japan Government Policy Evaluations Act*, the *U.S. Government Performance and Results Modernization Act*, and the *UK Local Government Act*-all of which regulate law enforcement evaluation. Currently, China’s evaluation initiatives are primarily reflected in practical achievements from Renmin University of China and the Chinese Academy of Social Sciences, along with certain policy documents, but lack formal legal recognition. To address this, China could adopt international best practices by formulating the *Implementation Measures for Administrative Action Evaluation*, establishing guiding ideology, principles, methodologies, and content standards for evaluating administrative actions-including intelligent approval-to promote systematic development of evaluation mechanisms and ensure standardized, scientific implementation of quality evaluation. However, despite the relative abundance of relevant legislation at present, the life of the law lies not in the grandeur or completeness of the legislative text, but in its actual operational effectiveness [84]. There is currently a lack of theoretical discussion on the legitimacy of law enforcement evaluation, resulting in an anti-logical situation of “institutions coming first, theories following behind,” which will weaken the authority and acceptability of the implementation of such laws [85]. This is particularly critical at a time when the focus is on good law and good governance as well as the construction of the rule of law, the legitimacy basis of law enforcement evaluation must be clarified from a theoretical perspective [86]. Given the multidisciplinary nature of law enforcement evaluation, this study proposes constructing a multi-layered framework for legitimacy analysis from an interdisciplinary perspective. For example, from the legal perspective, the principle of due process can be used to define the rule of law baseline of the evaluation process. From the public administration perspective, the theory of optimal administration can clarify the value objectives of law enforcement evaluation [87]. From the statistical perspective, the reliability and validity standards can be used to test the usability of the evaluation results. From the sociological perspective, the theory of deliberative democracy can be applied to give play to the rational participation of the public in the evaluation process [88]. Thus, by solidifying the theoretical foundation, the construction and implementation of relevant legal systems can achieve lasting vitality.

## 6. Conclusion

As a crucial component of digital administration and smart cities, intelligent approval has demonstrated remarkable efficiency advantages in service transformation. However, quality challenges of intelligent approval have emerged, affecting rationality, satisfaction, and supervision to varying degrees, which tightly link to government service. There is an urgent need to establish a a globally applicable quality evaluation indicator system to inspect and optimize intelligent approval. The Delphi Method and the “Value-Institution-Practice” framework can be adopted to design indicators focusing on legality, rationality, satisfaction, and supervision. The case study from Taiyuan through AHP and FCE has validated the practical applicability of this system, demonstrating its potential for broader implementation. The evaluation results show that the overall quality of intelligent approval of business licenses in Taiyuan is good, but there remain potential quality problems and prominent quality problems to be solved. The former includes insufficient specific modes in the approval neutrality, inaccessible the right to a hearing in the approval participation, inadequate staff service in the experience satisfaction and ineffective public supervision in the external supervision. The latter focuses on the lack of transparency. Accordingly, potential problems can be prevented from escalating into prominent problems by improving digital infrastructure, allowing the right to a hearing of post-decision where necessary, continuously enhancing the service competence of administrative staff, and refining public feedback mechanisms. And prominent problems can be addressed by establishing a multi-dimensional algorithm disclosure structure and providing algorithm explanations to ensure transparency in intelligent approval. The application of the intelligent approval quality evaluation indicator system has also revealed limitations in terms of organizational capacity, efficiency, authority, and standardization. Future research should focus on addressing these shortcomings. To this end, administrative organs should take the lead in establishing the dedicated evaluation committee, actively leverage digital platforms and computational programs, and promptly formulate guidelines or implementation rules for evaluating digital administration.

In the era of technology empowerment, digital administrative acts including intelligent approval still play an essential role in reducing transaction costs, creating a low-carbon environment, and enhancing government services. Intelligent approval just represents a microcosm of smart cities planning, yet their full potential in enhancing administrative efficiency and government service remains uncharted territory. What is certain, however, is that “people are the end goal, not the means.” Faced with institutional transformations brought by the digital age, administrative organs must redefine technology’s role as a service provider not a master, uphold the governance principle of “technology for good,” and steer urban planning from mere digitization toward digital services.
